# Population Genomics Reveals Gene Flow and Adaptive Signature in Invasive Weed *Mikania micrantha*

**DOI:** 10.3390/genes12081279

**Published:** 2021-08-20

**Authors:** Xiaoxian Ruan, Zhen Wang, Yingjuan Su, Ting Wang

**Affiliations:** 1 School of Life Sciences, Sun Yat-sen University, Guangzhou 510275, China; ruanxx@mail2.sysu.edu.cn (X.R.); wangzh535@mail2.sysu.edu.cn (Z.W.); 2 Research Institute of Sun Yat-sen University in Shenzhen, Shenzhen 518057, China; 3 College of Life Sciences, South China Agricultural University, Guangzhou 510641, China

**Keywords:** population genomics, gene flow, genotype–environment association, gene family, positive selection, invasive adaptation, *Mikania micrantha*

## Abstract

A long-standing and unresolved issue in invasion biology concerns the rapid adaptation of invaders to nonindigenous environments. *Mikania micrantha* is a notorious invasive weed that causes substantial economic losses and negative ecological consequences in southern China. However, the contributions of gene flow, environmental variables, and functional genes, all generally recognized as important factors driving invasive success, to its successful invasion of southern China are not fully understood. Here, we utilized a genotyping-by-sequencing approach to sequence 306 *M. micrantha* individuals from 21 invasive populations. Based on the obtained genome-wide single nucleotide polymorphism (SNP) data, we observed that all the populations possessed similar high levels of genetic diversity that were not constrained by longitude and latitude. *Mikania micrantha* was introduced multiple times and subsequently experienced rapid-range expansion with recurrent high gene flow. Using *F*_ST_ outliers, a latent factor mixed model, and the Bayesian method, we identified 38 outlier SNPs associated with environmental variables. The analysis of these outlier SNPs revealed that soil composition, temperature, precipitation, and ecological variables were important determinants affecting the invasive adaptation of *M. micrantha*. Candidate genes with outlier signatures were related to abiotic stress response. Gene family clustering analysis revealed 683 gene families unique to *M. micrantha* which may have significant implications for the growth, metabolism, and defense responses of *M. micrantha*. Forty-one genes showing significant positive selection signatures were identified. These genes mainly function in binding, DNA replication and repair, signature transduction, transcription, and cellular components. Collectively, these findings highlight the contribution of gene flow to the invasion and spread of *M. micrantha* and indicate the roles of adaptive loci and functional genes in invasive adaptation.

## 1. Introduction

Biological invasion has been reported at increasing rates and on expanding scales since the 20th century, gaining the attention of ecologists and evolutionary biologists [1]. A long-standing unresolved issue in invasion biology concerns the ability of invading species to rapidly adapt to novel environments in nonindigenous areas. A positive correlation between genetic diversity and invasion success has been proposed, as many invaders maintain a level of genetic diversity similar to or even higher than that of native species [2,3]. This correlation has been confirmed in many successful invasive species, especially those that have experienced severe genetic bottlenecks [4,5]. The maintenance of genetic diversity is often related to gene flow between invasive populations [6]. Gene flow can lead to genetic exchange between nearby populations and alter the distribution of genetic variation across the invaded range, preventing a decline in genetic diversity from core to edge populations [7]. Gene flow can also generate recombinant genotypes and new phenotypes following the introduction of divergent native source populations, providing additional invasion potential for the introduced populations [8,9]. In addition to the influence of genetic diversity and gene flow, adaptive response to invasive environments is considered another important mechanism promoting species’ survival and spread in new areas [10,11]. Species that successfully colonize new areas are or will be subject to environmental pressures and/or changes [12]. Changing environmental conditions leave adaptive signatures throughout the genomes of invasive species [13]. Evidence available to date indicates that genetic loci associated with environmental variables and many important functional genes are involved in the process of population expansion and the environmental adaptation of invasive species [14,15]. Hence, the elucidation of the effect of gene flow on genetic variation and the identification of genetic loci and functional genes responsible for invasive adaptation are important for the study of the population expansion and adaptation of alien species.

Early studies using anonymous markers (e.g., inter-simple sequence repeats (ISSRs) and amplified fragment length polymorphisms (AFLPs)) may have included bias in their assessment of genetic variation, while the utility of these markers to detect adaptive loci associated with environmental variables, especially when using few molecular markers, is limited. Furthermore, studies using anonymous markers cannot be used to determine the gene function of candidate loci. However, with the development of sequencing technology, reduced-representation genomic sequencing has provided an opportunity to obtain genome-wide sequence information across populations, greatly promoting research into population expansion and adaptive evolution in invasive plants [16]. Genotyping-by-sequencing (GBS) is a reduced-representation genome analysis method that identifies hundreds of thousands of single nucleotide polymorphism (SNP) markers across whole genomes [17]. As demonstrated by a large number of studies, the GBS approach is a useful and promising tool for researching invasive expansion and population adaptation. For example, Martin et al. (2016) [18] sequenced the genotypes of 190 *Ambrosia artemisiifolia* individuals from 37 locations and found that SNPs active in geographic differentiation occurred more often within pathways related to growth and defense responses than in other pathways. Van Boheemen et al. (2017, 2020) [4,15] analyzed *A. artemisiifolia* individuals from North America, Europe, and Australia, and found multiple introductions and adaptive signatures in response to environmental selection pressure. These studies have shown that the GBS approach can provide insight into the genetic basis underlying population invasion and contribute to the exploration of the environmental variables and genes involved in population adaptation.

*Mikania micrantha* H.B.K. (Asteraceae), a tropical American perennial creeping vine, is one of the top 10 most problematic noxious weeds worldwide [19,20]. The rapid expansion and invasion of this species in the Pacific Island and Southeast and South Asia regions, including southern China, has caused enormous economic losses and ecosystem damage [21,22]. The distribution of *M. micrantha* covers a relatively wide array of biome types, including tropical and subtropical moist and dry broadleaf forests, tropical and subtropical coniferous forests, tropical and subtropical grasslands, deserts and xeric shrublands, savannas, and temperate broadleaf and mixed forest [23]. *M. micrantha* plants can grow in a variety of soil types, in both dry and cold areas, in aquatic habitats, and in broad light niches [24,25,26]. The wide range of natural habitats of *M. micrantha* indicates its strong adaptability to different environmental conditions. The population of *M. micrantha* can not only adapt well to climate conditions similar to those of its native range, but also invade novel climatic niches [27]. Lineages with different genetic backgrounds occupy new climatic spaces in their respective invasive ranges [27]. The expansion and differentiation of *M. micrantha* into different climatic niches demonstrate its wide ecological amplitude and strong environmental adaptability. In addition, *M. micrantha* can propagate sexually via thousands of lightweight seeds [28]. Long-distance seed dispersal mediated by wind and human activities increases the genetic exchange between introduced populations, improving their invasion potential [21]. Frequent and long-distance seed dispersal may affect genetic differentiation and gene flow among *M. micrantha* populations. Considering its high dispersal potential and strong adaptability, *M. micrantha* is an ideal candidate for investigating the contribution of gene flow to population expansion, the response of genetic loci to environmental variables, and the role of functional genes in invasive adaptation.

*M. micrantha* spread rapidly throughout southern China following its introduction to the Hong Kong Zoological and Botanical Gardens in 1884 [29]. The role of genetic and epigenetic variations in the population expansion of *M. micrantha* in southern China has been investigated in several genetic studies, all of which used a small number of anonymous genomic markers [30,31,32,33,34,35]. Nevertheless, the available population genetic data failed to provide a comprehensive understanding of the genetic status and adaptive response of *M. micrantha* populations. Some key issues have not been resolved in these genetic studies. First, different molecular markers detected obviously different levels of genetic differentiation across populations distributed in similar geographic ranges (values ranging from 0.044 to 0.442), hindering the understanding of how gene flow contributes to the invasive success of *M. micrantha* in southern China. Secondly, genetic loci that respond to environmental variables have not been detected; consequently, the effect of environmental variables on genetic variation and population adaptation remains unresolved. Third, candidate functional genes related to colonization and population adaptation have not yet been identified. Population genomic data for natural populations of *M. micrantha* are needed to address these issues.

In the current study, we used the GBS approach to generate a large number of SNPs to analyze 306 *M. micrantha* individuals from 21 populations in six regions, covering the main part of its invasive range in southern China. Based on the SNP data, we detected the effect of gene flow on the genetic variation in the introduced populations of *M. micrantha*. We also identified outlier SNPs associated with environmental variables. Furthermore, the gene family clustering analysis of the genomes of *M. micrantha* and 12 other plants revealed unique gene families in *M. micrantha* that are important for adaptation. We also detected candidate genes that showed a significant positive selection signature in *M. micrantha* and explored their functions. The current study provides new insights into the population expansion and adaptation of *M. micrantha*.

## 2. Materials and Methods

### 2.1. Plant Material and Soil Sampling

**Figure 1:**
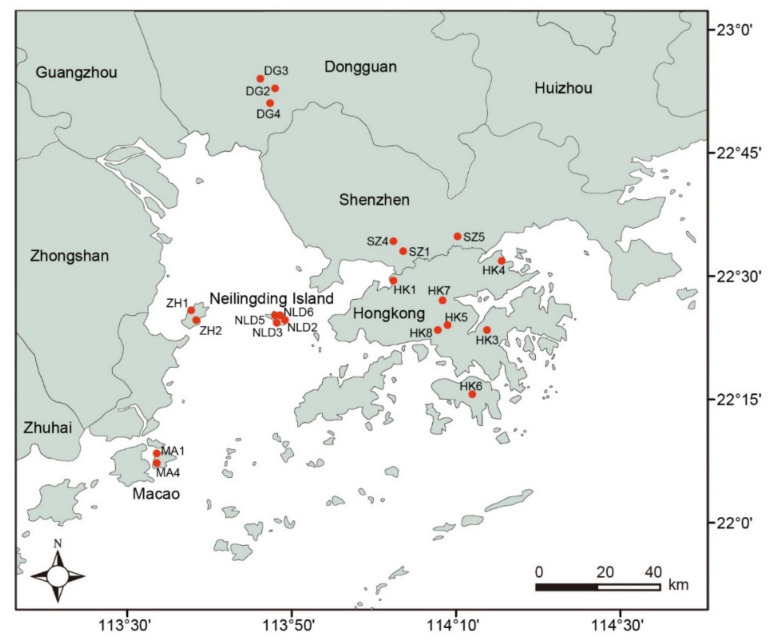
Sampling distribution of *M. micrantha* populations. The red dots represent 21 invasive populations from six regions, including Hong Kong, Macao, Shenzhen, Neilingding Island, Dongguan, and Zhuhai.

In 2016, 306 *M. micrantha* individuals were sampled from 21 invasive populations distributed in six regions in southern China, including Hong Kong, Macao, Shenzhen, Neilingding Island, Dongguan, and Zhuhai (Figure 1; Table S1). From each population, 10–16 plants each growing more than 15 m apart were randomly sampled. Fresh leaves were collected from each plant sample and preserved in silica gel. Additionally, three soil samples that were evenly distributed within each population were collected. Approximately 1 kg of soil per sample was taken from a depth below 10 cm.

### 2.2. DNA Extraction and GBS Library Sequencing

Genomic DNA from 306 *M. micrantha* individuals was extracted using the cetyltrimethylammonium bromide (CTAB) method [36]. DNA quality was evaluated using 1% agarose gel electrophoresis and the DNA amount was quantified using a NanoPhotometer spectrophotometer (IMPLEN, CA, USA). The DNA concentration was measured using a Qubit 2.0 Flurometer (Invitrogen, Life Technologies, CA, USA). High-quality DNA (concentration ≥ 20 ng/µL and mass ≥ 300 ng) was used for GBS library construction.

GBS libraries were constructed for 306 *M. micrantha* DNA samples using *Mse*Ι (New England Biolabs, NEB) and *EcoR*Ι restriction enzymes. Briefly, the DNA samples were incubated with *Mse*Ι at 37°C. The digested DNA samples were ligated to *Mse*Ι Y adapter N containing individual-specific barcodes with T4 DNA ligase. To further reduce the complexity and increase genome coverage, the *Mse*Ι-digested samples were redigested with *EcoR*Ι. *EcoR*Ι-digested DNA fragments were purified using Agencourt AMPure XP (Beckman) and PCR-amplified using Phusion Master Mix (NEB), universal primer, and index primer. Amplified DNA from 306 *M. micrantha* individuals was purified and screened for size. Size-selected fragments (insert sizes of 265–315 bp) were purified and diluted for sequencing. Samples for all *M. micrantha* individuals were divided into 12 pooled libraries, which were sequenced using the Illumina NovaSeq platform (Illumina, San Diego, CA, USA) to generate 150 bp paired-end reads.

### 2.3. Data Quality Control and SNP Calling

Sequences from each *M. micrantha* individual were sorted based on the individual-specific barcode within the raw reads. Raw reads for each individual were quality-controlled with in-house Perl scripts. Clean reads were obtained by removing reads that contained more than 10% ambiguous bases (N), more than 50% low-quality (Phered quality score < 5) bases, and bases that aligned to adapters exceeding 10 nt.

Clean reads from 306 individuals were aligned to the *M. micrantha* reference genome (unpublished genome data from our laboratory) using the Burrows-Wheeler Aligner (BWA) v0.7.17 [37] with the parameters mem -t 4 -k 32 -M. The alignment results were transformed into BAM files, which were then sorted in SAMtools v1.3 [38]. mpileup in SAMtools was used for SNP calling, with the parameters -q 1 -C 50 -t SP -t DP -m 2 -F 0.002. SNP quality control was conducted using BCFtools in SAMtools with the following command: vcfutils.pl varFilter -Q 20 -d 2 -D 100000. To ensure the reliability and accuracy of SNP identification, GATK v3.8 [39] was also used to identify SNPs, with the following parameters: -T VariantFiltration QD < 4.0, FS > 60.0, MQ < 40.0, and GQ < 5. Only SNPs identified by both SAMtools and GATK were retained. To obtain a reliable and high-quality SNP dataset, the SNP loci were filtered using the following criteria: genotype quality (GQ) > 20, depth of 5–100, minor allele frequency (MAF) > 0.05, call rate > 0.5, and only biallelic SNPs retained. To determine the locations and mutation types of SNPs in the *M. micrantha* genome, ANNOVAR [40] was used to annotate the high-quality SNPs identified in 306 individuals from 21 populations.

### 2.4. Genetic Variation and Population Structure

The genetic diversity indices, including the observed heterozygosity (*H*_O_), gene diversity (*H*_S_), allelic richness (*A*_R_), and inbreeding coefficients (*F*_IS_), were calculated for each population and region using the R package hierfstat [41]. The means and 95% confidence intervals of these four indices were also calculated, and the differences in these four diversity parameters among the six regions were tested using Kruskal–Wallis and post hoc Nemenyi test implemented in the R package PMCMRplus [42]. The number of private alleles for each population and region was determined using the R package poppr [43]. In addition, the correlation of population genetic diversity with the longitude and latitude was examined using linear regression and Pearson correlation analysis (*p* < 0.05).

To determine the level of genetic differentiation in the study areas, pairwise Weir and Cockerham’s *F*_ST_ values were assessed between populations/regions using the diveRsity package in R [44]. To test how geographic isolation affected the genetic differentiation of populations, the correlation between genetic and geographic distance was examined using the ade4 package in R [45]. Hierarchical analysis of molecular variance (AMOVA) was performed using the poppr package in R [43] to identify genetic variation partitioned within and among populations. The gene flow among populations/regions was also analyzed, and the parameter *N*_m_ was calculated from the pairwise *F*_ST_ values using the formula *N*_m_ = (1—*F*_ST_)/4*F*_ST_.

To investigate the genetic structure of *M. micrantha*, PLINK v1.9 [46] was used to filter the obtained high-quality SNP loci (see Section 2.3) using the following parameters: -indep-pairwise window size = 50, sliding window = 5, and correlation coefficient (r^2^) = 0.8. Filtered SNPs (5966 SNPs) were used for genetic structure analysis. ADMIXTURE v1.23 [47] was used to infer the population structure of 306 individuals. The number of genetic clusters (*K*) was set from 2 to 22. Cross-validation (CV) error was used to select the most likely number of clusters. The *K* value with the lowest CV error was determined to be the optimal number of clusters. GCTA v1.26 [48] was used to perform principal component analysis (PCA) to explore the genetic structure of *M. micrantha*. Based on the pairwise genetic distances among individuals, the unweight pair group method with the arithmetic mean (UPGMA) method implemented in MEGA v5.10 [49] was used to construct the phylogenetic tree. One thousand bootstrap replicates were conducted to obtain the branch support rate of the phylogenetic tree. The phylogenetic tree was visualized using FigTree v1.4.2 [50].

### 2.5. Environmental Variables

The environmental data comprised 19 bioclimatic variables, 25 soil factors, and 6 ecological variables. The bioclimatic variables corresponding to historic conditions (from 1960 to 1990) for each population were obtained from WorldClim v1.4 (http://www.worldclim.org (accessed on 5 March 2019)) at a 2.5-arc-minute resolution. The soil factors, including fresh water content; air-dried water content; pH; electrical conductivity; organic matter content; total N, C, K, Ca, Na, Mg, Al, P, S, Si, Fe, Mn, Zn, Cu, Pb, Cr, As, Se, Ni, and Cd were measured following the method of Shen et al. (2021) [35]. The ecological variables included altitude, normalized difference vegetation index, enhanced vegetation index, percent of tree cover, percent of non-tree vegetation cover, and percent of non-vegetation cover. Altitude values were determined from the sampling locations of the 21 populations. The remaining five ecological variables were acquired from the Moderate Resolution Imaging Spectroradiometer (MODIS) dataset stored in the Land Process Distributed Active Archive Center (http://lpdaac.usgs.gov (accessed on 8 March 2019), recorded in 2006–2016). The yearly mean value for each ecological variable derived from the MODIS dataset was computed using the maximum value composites function [51]. To reduce multicollinearity among environmental variables, the variance inflation factors (VIFs) were calculated for the 19 bioclimatic variables, 25 soil factors, and 6 ecological variables using the vif function implemented in R package usdm [52]. Environmental variables with VIF < 10 were retained, including five bioclimatic variables, 12 soil factors, and 6 ecological variables (Table S2).

### 2.6. Identification of Candidate Selective Loci and Function Annotation

To determine whether there were SNP loci potentially under selection in the 21 populations, an *F*_ST_-based outlier approach was used with the BayeScan software [53]. It has been reported that the BayeScan software has an advantage over other similar programs in reducing false-positive rates. This software employs a logistic regression that dissects genetic variation into a locus-specific *F*_ST_ coefficient (α) shared by all populations and a population-specific *F*_ST_ coefficient (β) shared by all loci [53]. A positive α value indicates diversifying selection, while a negative α value indicates purifying or balancing selection. BayeScan was run with the default parameters. To reduce false positives, only SNP loci with log_10_ (PO) > 2 were considered outlier SNPs under selection. Given that purifying or balancing selection is more prone to elevated false-positive rates when identifying outlier loci in BayeScan under the context of range expansion [54], only outlier SNPs with diversifying selection were analyzed. In the association analysis between outlier SNPs and environmental variables, only SNPs with positive α values were considered.

The genes containing outlier SNPs identified by BayeScan were functionally annotated. Annotation based on the Pfam protein database was performed using Hmmscan implemented in HMMER v3.1 [55]. Based on the annotation results in the Pfam database, gene ontology (GO) terms were acquired using Blast2GO [56] and a custom script. SwissProt database annotation was performed using DIAMOND v0.8.36 [57] with an E-value cutoff of 1 × 10^−5^.

### 2.7. Association of Candidate Selective Loci with Environmental Variables

The latent factor mixed model (LFMM) was used to detect the associations of outlier SNPs with environmental variables in the R package LEA [58]. LFMM has been confirmed to have a good balance between high power and a low false-positive rate, and thus to accurately detect the correlation of a single locus and a univariable [59]. The number of latent factors (*K*) was selected using the snmf function, which uses a Bayesian clustering algorithm to estimate the admixture coefficients of individuals. Based on the results of the snmf analysis obtained with 10 repetitions and 20,000 iterations for each *K* value from 1 to 22, the *K* value with the minimum cross-entropy was identified. This *K* value was used to perform LFMM analysis with the lfmm function. The lfmm function was implemented using 50,000 burn-in, 100,000 iterations, and 10 replicates. The *p*-values were used to test the significance of the correlation, with a *p*-value < 0.005 representing a significant association.

The Bayesian method implemented in the BAYENV v2.0 package [60] was also used to evaluate the association of allele frequencies with environmental variables. BAYENV uses the covariance of allele frequencies between populations as a null model to identify SNPs that are potentially under selection. To maximize the stability in estimating the null model, three covariance matrices were generated from three independent runs, and the number of iterations for each run was set to 100,000. The three covariance matrices were then averaged. To determine whether the average covariance matrix reflected the variance of allele frequencies between populations, the average covariance matrix was compared to a pairwise *F*_ST_ matrix using a Mantel test in the R package ade4 [45] with 1000 permutations. Based on the average covariance matrix, BAYENV was implemented three times to infer the Bayes factor for the correlation between each SNP and each environmental variable. SNP loci with a mean Bayes factor greater than three were retained as potentially undergoing selection.

### 2.8. Gene Family Analysis

Orthologous gene clusters in the genomes of *M. micrantha* and 12 other plants were identified using the OrthoMCL program [61]. In addition to the predicted *M. micrantha* proteins, protein sets from 12 other plants were obtained from public sources (Table S3). Protein sequences from the 13 species were processed for a minimum protein length of 30 amino acids. To obtain the similarity relationship between the protein sequences, an all-against-all comparison was conducted using BLASTP with an E-value cutoff of 1 × 10^−5^. OrthoMCL was used to cluster the BLASTP results to define gene families.

To identify biological pathways significantly enriched among the unique gene families of *M. micrantha*, Kyoto Encyclopedia of Genes and Genomes (KEGG) enrichment analysis was performed using KOBAS [62]. Adjusted *p*-values were used to determine the significance of the KEGG pathway enrichment, with adjusted *p*-values < 0.05 considered significant. In addition, ANNOVAR [40] was used to annotate the high-quality SNPs identified in 306 individuals from 21 populations to identify nonsynonymous SNPs. To further explore whether the genes unique to *M. micrantha* have developed nonsynonymous amino acid mutations at the population level, overlaps between genes containing nonsynonymous SNPs (Table S4) and genes unique to *M. micrantha* were detected. Overlapping genes were functionally annotated.

### 2.9. Identification of Positively Selected Genes

Based on the gene family clustering data for *M. micrantha* and the 12 other plants, single-copy orthologous genes of the 13 species were identified. The protein-coding sequences of single-copy orthologous genes were subjected to multiple sequence alignments using MUSCLE [63]. To identify candidate genes that underwent positive selection in *M. micrantha*, the branch-site model implemented in the codeml module of PAML [64] was used, with *M. micrantha* as the foreground branch and the 12 other plants as the background branches. The likelihood ratio test was used to evaluate the difference between the results obtained in the null and alternative models, and the *p*-values were tested again using the FDR method under the standard of 0.05. The positively selected genes (PSGs) were annotated and used for KEGG enrichment analysis. Furthermore, the overlaps between genes containing nonsynonymous SNPs and PSGs in *M. micrantha* were detected.

## 3. Results

### 3.1. GBS Sequencing and SNP Calling

GBS library sequencing of 306 *M. micrantha* individuals from 21 invasive populations was performed. The analysis yielded a total of 3320.63 million raw reads, with a mean of 10.85 million reads per individual (Table S5). After quality filtering, 3320.39 million clean reads representing 478.14 G bases were retained, with a mean Q20 of 95.01% and a mean Q30 of 88.16%. The clean reads for each individual were mapped to the *M. micrantha* reference genome. The individual mapping rates ranged from 85.65% to 98.42% (Table S6), indicating good mapping. We successfully identified 117,870 and 5,066,387 SNPs using GATK and SAMtools, respectively. To ensure the reliability of the SNP identification, only SNPs identified by both GATK and SAMtools were selected. A total of 54,392 SNPs were simultaneously identified by these two methods. After filtering, 16,944 high-quality SNPs were identified across 306 individuals, with an average SNP call rate of 90.03% (range 42.94–96.88%) (Table S7). Among the 16,944 identified SNPs, 10,207 SNPs were involved in transition mutations and 6737 in transversion mutations, with the most common mutation type being a C/T transition (5156, 30.43%) and the least common being a C/G transversion (1089, 6.43%) (Table S8). Furthermore, most of the SNPs were located in intergenic regions (13,984, 82.53%), with only 5.04% (854) located in exonic regions (Table S9). A total of 402 synonymous SNPs and 305 nonsynonymous SNPs were located within exons, generating a nonsynonymous/synonymous ratio of 0.76.

### 3.2. Genetic Variation and Population Genetic Structure

A similar genetic diversity was found in the 21 studied populations, with allelic richness (*A*_R_) varying from 1.743 (HK3) to 1.886 (HK1), observed heterozygosity (*H*_O_) ranging from 0.280 (HK4) to 0.387 (DG4), and gene diversity (*H*_S_) ranging from 0.363 (MA1) to 0.458 (SZ1) (Table 1). The inbreeding coefficient (*F*_IS_) values were positive in all 21 populations. The distribution of genetic diversity across 21 populations was not limited by longitude and latitude (*p* > 0.05), except for a weak correlation between *A*_R_ and latitude (Figures S1 and S2). In addition, no private alleles were found across populations. All six regions were consistently found to have similar genetic diversity and positive *F*_IS_ values. The Kruskal–Wallis and post hoc Nemenyi test demonstrated that there were no significant differences in *A*_R_, *H*_S_, *H*_O_, and *F*_IS_ between the six regions (*p* > 0.05) (Table S10).

Regarding population differentiation, the *F*_ST_ value was highest between HK3 and NLD2 (0.133) and lowest (0.004) between NLD2 and NLD6 (Table S11). We further evaluated the pairwise *F*_ST_ values among the six regions, with *F*_ST_ ranging from 0.008 (SZ versus DG) to 0.043 (SZ versus NLD) (Table S12). These results indicated low levels of genetic differentiation in *M. micrantha*, implying that gene flow between populations and between regions was common. Indeed, we detected a high rate of gene flow between populations and between regions. The level of gene flow (*N*_m_) between populations ranged from 1.63 to 62.25 and from 5.564 to 31 between regions (Tables S11 and S12).

**Figure 2:**
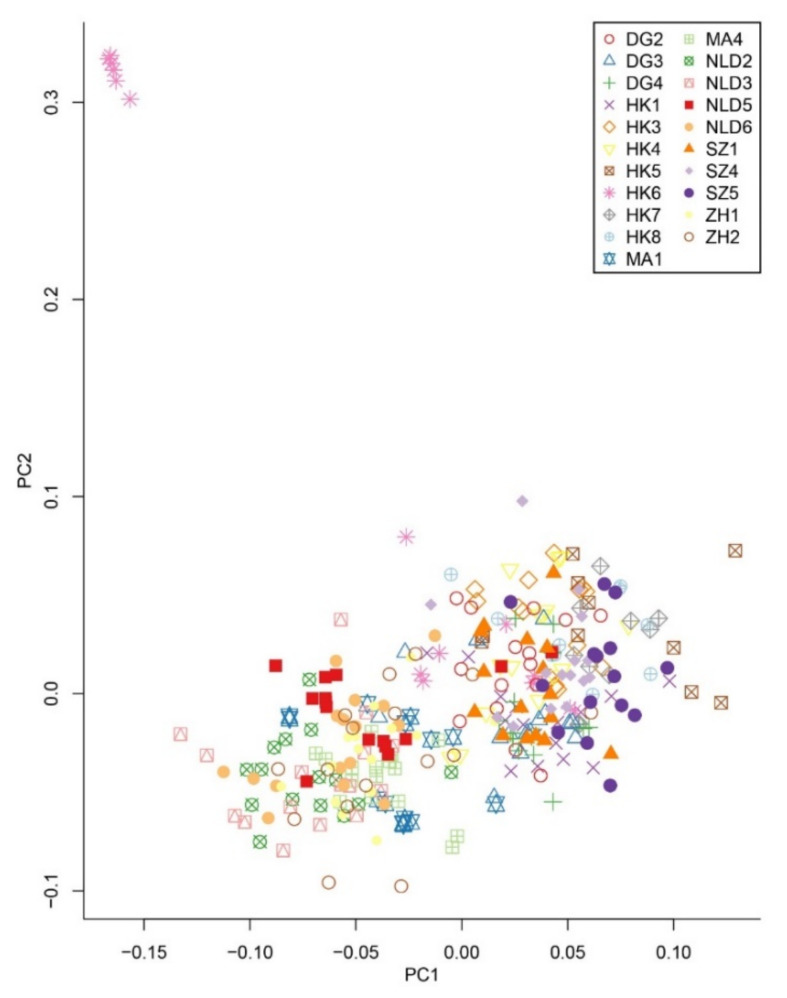
Principal component analysis (PCA) of the 306 *M. micrantha* individuals from 21 invasive populations. The top two principal components (PC1 and PC2) explained 4.022% and 2.526% of the total genetic variation, respectively.

**Figure 3:**
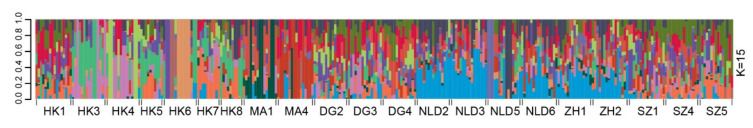
Population genetic structure analysis of the 306 *M. micrantha* individuals inferred from the software ADMIXTURE. The height of each colored column represents the proportion of individual assigned to different genetic clusters.

Principal component analysis (PCA) revealed an admixed distribution pattern in which individuals from different populations clustered randomly (Figure 2). To further explore the population structure of *M. micrantha*, genetic clustering of the 306 individuals was conducted using ADMIXTURE, which also provided evidence of an admixed structure at the optimal clustering level (*K* = 15) (Figure 3 and Figure S3). Consistent with the results from the ADMIXTURE and PCA analyses, the UPGMA tree also reflected an unclear genetic relationship within *M. micrantha*. Individuals within a population were allocated to different genetic clades of the phylogenetic tree, while individuals from distinct populations were located on the same branch (Figure S4).

The analysis of molecular variance (AMOVA) revealed that 95.84% of the total variation occurred within populations, while only 4.16% occurred among populations (*p* < 0.001, Table S13). AMOVA detected a low genetic differentiation among populations (*F*_ST_ = 0.042; *p* < 0.001). The Mantel test showed that the genetic differentiation between populations included a weak genetic relatedness caused by isolation by distance (IBD) (r = 0.311, *p* = 0.006).

### 3.3. Identification of Candidate Selective Loci and Gene Annotation

**Figure 4:**
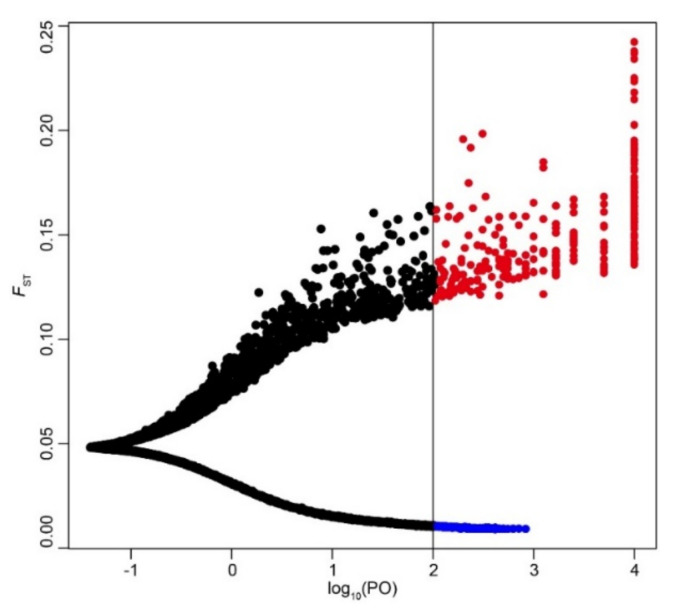
The scatter plot from Bayesian outlier analysis of SNPs. The vertical black line shows the threshold of log_10_ (PO) = 2, and SNP loci with log_10_ (PO) > 2 were considered outlier SNPs. The blue and red circles represent the outlier SNPs with negative and positive α values, respectively.

In the BayeScan analysis, 273 SNPs with diversifying selection were considered to be outlier SNPs at a threshold of log_10_ (PO) > 2 (Figure 4). These 273 outlier SNPs might be under selection, which would be of great significance to the invasion and adaptation of the 21 populations analyzed. The *F*_ST_ values of the 16,944 SNPs evaluated by BayeScan ranged from 0.009 to 0.242, with a mean value of 0.05. Over 96% of the SNPs (16,321 of 16,944; 96.32%) showed an *F*_ST_ < 0.1. Outlier SNPs also exhibited low *F*_ST_ values, with a mean value of 0.151, implying that the 21 populations did not show significant adaptive differentiation at the outlier loci. The outlier SNPs were located in different regions of the *M. micrantha* genome, with 11 outlier SNPs residing in exon regions of 11 genes (Table S14). The 11 genes had a length ranging from 5714 to 16,509 bp (Table S15). In addition, these 11 genes were annotated in the SwissProt and Pfam protein databases. GO terms were used to functionally classify the 11 genes. The genes were assigned to 29 terms: 10 terms in “biological process”, 8 terms in “cellular component”, and 11 terms in “molecular function” (Table S15). Under the biological process category, “metabolic process” (GO: 0008152), “response to stimulus” (GO: 0050896), and “oxidation–reduction process” (GO: 0055114) were highly representative GO terms. For the molecular function category, the most abundant functions were related to “binding” (GO: 0005488), “transporter activity” (GO: 0005215), and “transcription regulator activity” (GO: 0140110). In terms of the cellular component category, “membrane” (GO: 0016020) and “membrane part” (GO: 0044425) were the most noticeable functions.

### 3.4. Association of Candidate Selective Loci with Environmental Variables

We utilized two outlier test approaches—a LFMM analysis and the Bayesian method implemented in BAYENV—to detect signatures of adaptation among *M. micrantha* populations. These two approaches have great power for the identification of outlier loci associated with environmental variables. We identified 31 associations between 18 outlier SNPs and six environmental variables in the LFMM analysis (Table S16), and 67 associations between 24 outlier SNPs and eight environmental variables in BAYENV (Table S17). Combining the results of the two analysis approaches, we detected 96 associations between 38 outlier SNPs and 13 environmental variables. Among the associations, 14 were related to temperature, 25 to precipitation, 25 to soil factors, and 6 to ecological variables. Of the associated environment variables, soil C content was associated with the most outlier SNPs (21), followed by mean temperature of driest quarter (14), isothermality (12), precipitation of driest month (12), annual precipitation (11), precipitation of warmest quarter (7), soil Si content (6), soil Ca content (5), and percent of non-vegetation cover (4). Besides these nine environmental variables, soil K content and percent of tree cover were also noteworthy factors, and these may play a potential role in the adaptation of *M. micrantha*. Notably, four outlier SNPs were simultaneously identified in the BayeScan, LFMM, and BAYENV analyses and were associated with 10 environmental variables, suggesting that environmental variables are important factors affecting the population adaptation of *M. micrantha*.

### 3.5. Gene Family Analysis

**Figure 5:**
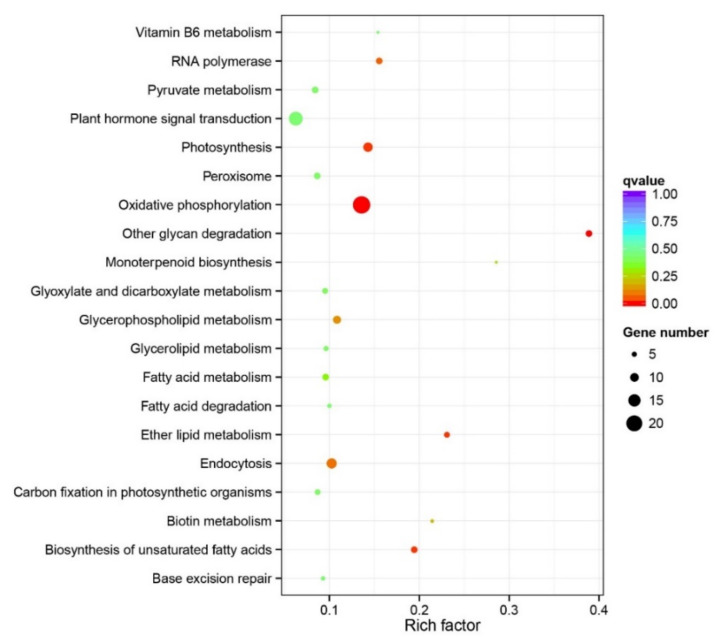
Kyoto Encyclopedia of Genes and Genomes (KEGG) pathway enrichment analysis of 683 gene families unique to *M. micrantha*.

To explore the special roles of unique gene families in the expansion and adaptation of *M. micrantha*, we performed a gene family clustering analysis on the genomes of *M. micrantha* and 12 other plants. Using the predicted protein set of these plants, a total of 36,638 orthologous gene families consisting of 409,166 genes were identified (Table S18). Furthermore, 6346 gene families containing 170,110 genes shared among the 13 plants and 683 gene families comprising 2256 genes were found to be unique to *M. micrantha* (Tables S18–S20). The length of the 2256 genes unique to *M. micrantha* ranged from 154 to 122,119 bp (Table S19). Among the gene families unique to *M. micrantha*, KEGG enrichment analysis revealed five significantly overrepresented KEGG pathways—“oxidative phosphorylation” (ko00190), “biosynthesis of unsaturated fatty acids” (ko01040), “photosynthesis” (ko00195), “other glycan degradation” (ko00511), and “ether lipid metabolism” (ko00565)—all of which are important for the growth, metabolism, and defense response of *M. micrantha* (Figure 5; Table S21).

Further, we found that eight genes unique to *M. micrantha* have developed mutations resulting in nonsynonymous amino acid substitutions across 21 populations. Among the eight candidate genes, three, four, and five genes were annotated in the SwissProt, Pfam, and GO databases, respectively (Table S22). These candidate genes encode proteins and participate in some functional categories such as glutathione *S*-transferase T3 (GSTT3) and “integral to membrane” (GO: 0016021), respectively.

### 3.6. Positive Selection Gene Analysis

The gene family clustering analysis revealed 393 single-copy orthologous genes among *M. micrantha* and the 12 other plants. Following the likelihood ratio test, 41 genes were considered candidate genes that had undergone positive selection in *M. micrantha* (Table S23). The 41 genes had a length ranging from 1723 to 16,686 bp (Table S24). Of the 41 PSGs, 30, 33, and 38 PSGs were annotated in the SwissProt, Pfam, and GO databases, respectively (Table S24). In the GO annotation, the highly represented GO terms were related to the functions of (i) binding, (ii) DNA replication and repair, (iii) signature transduction, and (iv) transcription and cellular components (Figure S5). These functions may be important for the colonization and adaptation of *M. micrantha*. KEGG enrichment analysis of 41 PSGs showed that “SNARE interactions in vesicular transport” (ko04130) were significantly enriched (Table S25; Figure S6). In addition, two PSGs harbored mutations resulting in nonsynonymous amino acid substitutions in the 21 *M. micrantha* populations, and these were mainly related to binding, membrane component, and fatty acid biosynthesis.

## 4. Discussion

### 4.1. Population Variation and Structure

During population invasion, factors affecting the invasiveness of alien species, such as genetic diversity, may be influenced by genetic events, geographic distance, gene flow, and human activities [65]. Alien species that colonize nonindigenous areas generally experience the founder effect or bottleneck events that lead to a reduction in genetic diversity and influence the invasive potential of the species populations [66]. Heterozygosity and allele diversity are two representative indices of genetic diversity that are sensitive to the founder effect and bottleneck events [67,68]. In this study, obvious heterozygote deficiency was detected in *M. micrantha* across populations and regions. This is understandable, considering that *M. micrantha* has experienced severe founder effect and genetic bottlenecks during rapid colonization and invasion [33,69]. Inbreeding is a genetic trait observable in some invasive species [4] that can give rise to heterozygote deficiency within populations [70]. As *M. micrantha* is a self-incompatible species [71] and its local spread mainly relies on clonal propagation [72], inbreeding may be an important factor contributing to heterozygote deficiency. Indeed, we observed positive *F*_IS_ values in all populations and regions, suggesting the occurrence of inbreeding in the studied populations and regions.

The genetic differentiation levels in *M. micrantha* were quite low (0.004 ≤ *F*_ST_ ≤ 0.133), and lower than the interpopulation genetic differentiation level reported by previous genetic studies based on a small number of molecular markers (8–483 markers) [30,31,34]. In general, low-resolution molecular markers may cause bias in the assessment of genetic patterns. The genomic SNP data presented herein improved the assessment of genetic differentiation among populations of *M. micrantha*. Low genetic differentiation levels may occur because *M. micrantha* plants can produce enormous numbers of lightweight seeds that are dispersed over long distances by the wind [21], promoting colonization at different locations. The local establishment of *M. micrantha* mainly depends on clonal propagation, which may also be an important factor leading to low genetic differentiation. Human activities can result in seeds or cloned fragments being carried to different locations, increasing genetic exchange between individuals from different populations and weakening population divergence. In addition, the homogenization effect of frequent gene flow would reduce adaptive differentiation between populations. Consistent with this, we detected a high rate of gene flow (1.63 ≤ *N*_m_ ≤ 62.25) among *M. micrantha* populations. Similarly, a high rate of gene flow has been found among populations within the Asian invasion range [33], indicating that *M. micrantha* population invasion is accompanied by a high rate of gene flow.

Generally, the range limit linked to species invasions affects the genetic diversity of invasive populations [73]. The genetic diversity of invasive populations generally decreases from the abundance centers where it was first introduced to the range margin [73]. However, marginal populations often rely on central or nearby populations as genetic sources for colonization, persistence, and range expansion [7,74]. A high rate of gene flow can release invasive species from environmental and geographical constraints on genetic diversity [5,6]. In addition to gene flow, multiple introductions can also lead to increased genetic diversity through recombination between invasive genotypes [5]. In this study, all populations were found to possess high and similar levels of genetic diversity that were higher than those detected previously in invasive and native *M. micrantha* populations [31,34,75], higher than those reported for other invasive plants [4,76], and not limited by longitude and latitude across the entire invasive area. These results suggest that *M. micrantha* exhibits patterns of population genetics that tend to enhance its invasion potential and that gene flow and multiple introductions (see the following paragraph) may mitigate the negative influence of the founder effect, genetic bottleneck, or inbreeding on genetic diversity and mediate genetic diversity distribution in its invasive range.

ADMIXTURE and PCA analysis revealed multiple genetic clusters and an admixed population structure in the study area, which was consistent with the genetic structure previously detected in populations that invaded Asia [33,69]. Multiple introduction and a high rate of gene flow are two important driving factors that cause population admixture. Generally, invasive populations that have undergone multiple introductions often contain private alleles from different geographic sources [76]. No private alleles were detected among the populations assessed in our study. We speculate that frequent gene flow likely mediates genotype movement among populations that are occupied by different native genotypes. In fact, a high rate of gene flow was observed among populations herein, which may constrain adaptive divergence and lead to a weak population structure [77]. The influence of gene flow on the genetic structure of *M. micrantha* was also confirmed by the weak correlation between genetic differentiation and geographic distance. Geographical isolation can explain genetic differentiation during the early period of population invasion, when populations are geographically dispersed. The effect of geographical isolation on population differentiation becomes weaker with a high rate of gene flow during population expansion. The continued invasion of *M. micrantha* in southern China (more than 130 years) and high rate of gene flow among populations can explain the weak correlation between genetic differentiation and geographic distance. Overall, *M. micrantha* was introduced multiple times and subsequently experienced a rapid range expansion with recurrent high rates of gene flow.

### 4.2. Adaptive Response to Environmental Variables

The detection of association between SNPs and environmental variables helps us to recognize the bioclimatic, soil, and ecological variables that contribute to population adaptation. During the process of *M. micrantha* invasion from tropical America to southern China, the new environments encountered likely induced candidate-selected loci that respond to environmental conditions. The LFMM and BAYENV analysis revealed significant associations between 38 outlier SNPs and 13 environmental variables, including temperature, precipitation, soil, and ecological variables, all of which may play roles in the invasive adaptation of *M. micrantha*. Specifically, (i) rapid adaptation to climate is conducive to range expansion of invasive plants. Temperature and precipitation are important determinants of the growth, adaptation, and potential distribution of *M. micrantha* [23]. Consistent with this, the mean temperature of driest quarter, isothermality, precipitation of driest month, annual precipitation, and precipitation of warmest quarter were significantly associated with outlier SNPs, which reflects the importance of these environmental variables in the distribution of this species [35,78] and indicates that the *M. micrantha* genome responds to temperature and precipitation fluctuations. (ii) The significant effect of soil composition on the invasion of *M. micrantha* has been confirmed [79]. The soil C/Ca/K/Si content was found to be significantly associated with outlier SNPs. These findings are not unexpected, considering the functional importance of these soil factors. For instance, C supply has an important effect on the expression of genes involved in primary and secondary metabolism, growth, transport, signaling, and defense [80]. C depletion has been found to be related to drought-induced seed abortion in maize [81]. Ca plays a crucial role in regulating plant growth, development, and stress responses [82]. Furthermore, K is an essential nutrient in plants and its deficiency alters plant growth, photosynthetic performance, and antioxidant capacity [83]. Indeed, soil K availability regulates plant invasive success [84] and it has been reported that the rapid growth of *M. micrantha* depends on accelerated activation of soil K [85]. Finally, Si participates in various physiological processes—e.g., it increases the chlorophyll biosynthesis and assimilation rate and alleviates oxidative damage in stressed plants [86]. In addition, Si supply can increase K accumulation in the shoots [86]. (iii) Ecological variables are also important factors affecting the adaptation of *M. micrantha*. We found significant associations between outlier SNPs, the percentage of non-vegetation cover, and the percentage of tree cover. *M. micrantha* often forms a dense thicket above nearby plants [21] and escapes the negative effects of tree or other vegetation cover on light availability [87]. These correlations suggest that the *M. micrantha* genome may respond positively to tree or vegetation cover. Taken together, these results reveal that the *M. micrantha* genome already has selective signatures that respond to the changing environment to promote its invasive adaptation.

### 4.3. Genes Unique to M. micrantha May Be Important for Adaptation

Based on gene family clustering analysis, we identified gene families unique to *M. micrantha*. Unique gene families were also observed in the genomes of *Artemisia annua* [88] and *Chrysanthemum nankingense* [89]. Gene families unique to *M. micrantha* were enriched in functional pathways related to important physiological processes and stress response, such as oxidative phosphorylation, the biosynthesis of unsaturated fatty acids, and photosynthesis. Oxidative phosphorylation is an important metabolic pathway that generates ATP required for plant growth and metabolism. There is evidence that oxidative phosphorylation plays a crucial role in abiotic stress responses. For instance, oxidative phosphorylation is enriched by genes associated with the cold tolerance in *Anthurium andraeanum* [90]. Unsaturated fatty acids play crucial roles in multiple biological processes, enabling the plant to respond to biotic and abiotic stress [91]. High photosynthesis capacity is a representative feature of *M. micrantha*, providing the energy supply needed for rapid vegetative growth [92]. The enrichment of unique gene families in the photosynthesis pathway reflects the importance of photosynthesis in the invasion and adaptation of *M. micrantha*. Similarly, the upregulation of genes related to photosynthesis promotes the adaptation of *Solidago canadensis* to a shaded habitat, greatly improving the plant’s ability to adapt to different light conditions [93]. The identification of unique gene families in *M. micrantha* with functional importance will provide a valuable resource for further research on the adaptive mechanisms of this species.

Unique genes that harbor mutations leading to nonsynonymous amino acid substitutions among populations are worthy of attention. These unique genes encode functional proteins and are involved in important function categories. For example, GSTT3 is a member of GST superfamily and is related to antioxidants that respond to drought and biotic stress [94,95]. We detected a unique gene encoding the GSTT3 protein, which may have significant implications for increasing the tolerance of *M. micrantha* to drought and biotic stress. We also found unique genes related to ‘integral to membrane’. This functional category is related to the defense response of apple root to pathogen infection [96]. Although these genes were not identified as candidate loci for selection, they may become loci with an adaptive importance after sufficient time.

### 4.4. Role of Adaptive Genes in M. micrantha

Genes under evolutionary pressure (such as positive selection) may be important for the survival and spread of various species [97]. The identification of functionally important positive selection genes for *M. micrantha* would enable us to better understand the molecular associations underpinning its invasive adaptation. Accordingly, we identified 41 genes showing a significant positive selection signature in *M. micrantha*. These PSGs were found to be involved in (i) binding, (ii) DNA replication and repair, (iii) signature transduction, and (iv) transcription and cellular components (Figure S5; Table S24). Function binding, including DNA binding, ATP binding, and GTP binding, generally participates in various physiological processes and stress responses. For example, DNA/ATP/GTP-binding proteins play important regulatory roles in the response to cold stress in mature pollen of *Arabidopsis*, and in the response to heat stress during the imbibition and germination of *Ricinus communis* seed [98,99]. DNA replication is indispensable for the growth, development, and proliferation of cells in plants. A growing body of research shows that minichromosome maintenance (MCM) complex proteins are important regulatory factors essential for DNA replication. The overexpression of a single subunit of pea MCM6 improves salt tolerance in transgenic tobacco plants [100]. Plants have also developed a variety of mechanisms to repair DNA damage caused by abiotic stresses. Casati and Walbot (2008) showed that the DNA excision repair protein ERCC-1 (ERCC1) enhances the efficiency of UV-B-induced DNA damage repair in maize [101]. Exonuclease 1 (Exo1) is known to function in a number of DNA repair pathways [102]. The positive selection of genes encoding MCM6, ERCC1, and Exo1 might be an adaptive response allowing *M. micrantha* to adapt to invasive environments under various abiotic stresses. Signal transduction links the sensing mechanism with genetic responses, which is important for signal transmission to the cellular machinery to initiate adaptive responses upon an environmental change [103]. For example, the G-protein-coupled receptor signaling pathway participates in cold tolerance in hullless barley (*Hordeum vuglare*) [104]. This pathway may have important implications for the response of *M. micrantha* to cold stimulus. Finally, key cellular components (e.g., nucleus) and transcriptional regulation are essential for plant growth and environmental adaptation. The positive selection of genes related to the nucleus and transcription suggests their potential roles in *M. micrantha*. In addition, two functionally important PSGs harbored mutations that resulted in nonsynonymous amino acid substitutions among 21 populations, which implies that they may contribute to the population adaptation of *M. micrantha*.

During rapid population invasion, genes that regulate important biological processes are expected to function in *M. micrantha*, providing insights into *M. micrantha* adaptation. We detected 11 outlier SNPs distributed in 11 genes as candidate targets of adaptive importance. The GO annotation analysis of the 11 genes revealed that they were related to stress response, which should be considered in future population genomic research. Under the biological process category, “metabolic process”, “oxidation–reduction process”, and “response to stimulus” were highly representative GO terms important for the environmental adaptation of invasive and non-invasive plants. The metabolic process may contribute to the cold tolerance in the northward invasion of *Alternanthera philoxeroides* [105]. The oxidation–reduction process is involved in the drought stress response in *Amorpha fruticosa* seedlings [106] and in the adaptation to hypoxia and low temperatures in *Kandelia obovata* [107]. Effective responses to environmental changes or stress stimuli are beneficial for the colonization and adaptation of invasive plants in nonindigenous areas. Response to stimulus is considered an important adaptation mechanism for plants under stress, e.g., due to water deprivation [108]. In the molecular function category, GO terms such as “binding”, “transporter activity”, and “transcription regulator activity” are generally related to stress responses, and have been shown to participate in the K deficiency response in tobacco [109] and the salt resistance of the invasive plant *Phragmites karka* [110]. In terms of the cellular component category, the membrane is an important structure that indirectly or directly perceives stimulation to initiate signal transduction. For instance, several membrane genes are upregulated in *Tamarix ramosissima* in response to water deficiency [111].

## 5. Conclusions

In the present study, we used population genomics to characterize the range expansion and invasive adaptation of *M. micrantha* in southern China. Genome-wide SNP analysis revealed, for the first time, the effects of gene flow, environmental variables, and functional genes on the population variation and adaptation of *M. micrantha*. The presented findings demonstrate the importance of a high rate of gene flow coupled with genetic admixture for the invasion success of *M. micrantha*. We identified 38 outlier SNPs associated with environmental variables. Soil composition, temperature, precipitation, and ecological variables were important determinants affecting the invasive adaptation of *M. micrantha*. Candidate genes with outlier signatures were related to abiotic stress responses. Gene family clustering analysis revealed 683 gene families unique to *M. micrantha* that may have significant implications for the growth, metabolism, and defense response in *M. micrantha*. Forty-one genes with significant positive selection signatures were identified. These PSGs mainly function in binding, DNA replication and repair, signature transduction, transcription, and cellular components. In addition, eight genes unique to *M. micrantha* and two PSGs harbored mutations leading to nonsynonymous amino acid substitutions across 21 populations; these may play crucial roles in the population expansion and adaptation of *M. micrantha*. Overall, the presented population genomic analysis indicates that the sustained high rate of gene flow, response of adaptive loci, and regulation of functional genes have enabled the invasion and adaptation of *M. micrantha* in southern China. These findings can be used to guide prevention and monitoring efforts for this invasive species.

## Figures and Tables

**Table 1:** Overview of genetic diversity indices within invasive populations and regions of *M. micrantha*. The genetic diversity indices represent the mean (±95% confidence) values of genotyped individuals.

Population/Region	*A*_R_	*H*_O_	*H*_S_	*F*_IS_
HK1	1.886 (0.003)	0.335 (0.003)	0.374 (0.003)	0.084 (0.006)
HK3	1.743 (0.005)	0.293 (0.004)	0.364 (0.003)	0.203 (0.007)
HK4	1.772 (0.005)	0.280 (0.003)	0.424 (0.003)	0.364 (0.006)
HK5	1.770 (0.005)	0.309 (0.004)	0.374 (0.003)	0.151 (0.007)
HK6	1.787 (0.005)	0.297 (0.004)	0.407 (0.003)	0.278 (0.007)
HK7	1.835 (0.005)	0.379 (0.004)	0.427 (0.003)	0.104 (0.007)
HK8	1.783 (0.005)	0.335 (0.004)	0.392 (0.003)	0.142 (0.007)
HK	1.983 (0.001)	0.315 (0.003)	0.411 (0.002)	0.247 (0.005)
SZ1	1.860 (0.004)	0.323 (0.003)	0.458 (0.003)	0.298 (0.006)
SZ4	1.875 (0.004)	0.362 (0.004)	0.426 (0.003)	0.146 (0.006)
SZ5	1.857 (0.004)	0.336 (0.004)	0.371 (0.003)	0.093 (0.006)
SZ	1.986 (0.001)	0.340 (0.003)	0.428 (0.002)	0.213 (0.005)
DG2	1.852 (0.004)	0.317 (0.003)	0.448 (0.003)	0.277 (0.006)
DG3	1.850 (0.004)	0.336 (0.003)	0.391 (0.003)	0.127 (0.006)
DG4	1.877 (0.004)	0.387 (0.004)	0.430 (0.003)	0.103 (0.007)
DG	1.979 (0.001)	0.347 (0.003)	0.429 (0.002)	0.196 (0.005)
NLD2	1.786 (0.005)	0.297 (0.004)	0.380 (0.003)	0.217 (0.007)
NLD3	1.826 (0.004)	0.325 (0.004)	0.394 (0.003)	0.177 (0.006)
NLD5	1.820 (0.004)	0.317 (0.004)	0.397 (0.003)	0.195 (0.007)
NLD6	1.842 (0.004)	0.308 (0.003)	0.396 (0.003)	0.208 (0.006)
NLD	1.965 (0.002)	0.312 (0.003)	0.396 (0.003)	0.227 (0.005)
ZH1	1.796 (0.005)	0.292 (0.003)	0.443 (0.003)	0.339 (0.006)
ZH2	1.852 (0.004)	0.330 (0.003)	0.419 (0.003)	0.223 (0.006)
ZH	1.952 (0.002)	0.312 (0.003)	0.435 (0.003)	0.297 (0.005)
MA1	1.790 (0.005)	0.333 (0.004)	0.363 (0.003)	0.091 (0.007)
MA4	1.811 (0.005)	0.321 (0.004)	0.414 (0.003)	0.223 (0.007)
MA	1.930 (0.003)	0.327 (0.003)	0.404 (0.003)	0.197 (0.006)

*A*_R_, allelic richness; *H*_O_, observed heterozygosity; *H*_S_, gene diversity; *F*_IS_, inbreeding coefficient.

## Data Availability

All sequencing data in this study has been deposited in the Sequence Read Archive (SRA) database under BioProject accession PRJNA656909.

## Supplementary Materials

The following are available online at https://www.mdpi.com/article/10.3390/genes12081279/s1, Figure S1: Correlation between genetic diversity indices and longitude of sampling populations. Diversity indices include allelic richness (*A*_R_), observed heterozygosity (*H*_O_), gene diversity (*H*_S_), and inbreeding coefficient (*F*_IS_), Figure S2: Correlation between genetic diversity indices and latitude of sampling populations. Diversity indices include allelic richness (*A*_R_), observed heterozygosity (*H*_O_), gene diversity (*H*_S_), and inbreeding coefficient (*F*_IS_), Figure S3: The optimum number of clusters (*K*) estimated with ADMIXTURE analysis based on cross-validation (CV) error, Figure S4: Unweight pair group method with arithmetic mean (UPGMA) tree constructed using the pairwise genetic distances among 306 *M. micrantha* individuals, Figure S5: Gene ontology (GO) annotation of the positive selection genes in *M. micrantha*. The red, orange, and green bars represent cellular component, biological process, and molecular function categories, respectively, Figure S6: Kyoto Encyclopedia of Genes and Genomes (KEGG) pathway enrichment analysis of the positive selection genes in *M. micrantha*, Table S1: Sampling description of 306 *M. micrantha* individuals and soil samples from 21 invasive populations in southern China, Table S2: The environmental variables with variance inflation factors (VIFs) < 10 for 21 *M. micrantha* populations, Table S3: Versions and download links of genomes from 12 other plants, Table S4: Information of SNPs showing nonsynonymous amino acid mutations in 306 *M. micrantha* individuals, Table S5: Statistic results of sequencing quality for the 306 *M. micrantha* individuals, Table S6: The mapping rates between the clean reads of each individual and the reference genome, Table S7: Number of SNPs identified across 306 *M. micrantha* individuals, Table S8: Summary of transition and transversion mutations of single nucleotide polymorphisms (SNPs) identified across 306 *M. Micrantha*, Table S9: The locations and mutation types of SNPs in the *M. micrantha* genome, Table S10: The analysis results of the differences in genetic diversity parameters among the six regions obtained from Kruskal–Wallis and post hoc Nemenyi test, Table S11: Genetic differentiation and gene flow between pairwise populations for *M. micrantha* individuals. The lower triangle represents genetic differentiation values (*F*_ST_), and the upper triangle represents gene flow between pairwise populations, Table S12: Genetic differentiation and gene flow between pairwise regions for *M. micrantha*. The lower triangle represents genetic differentiation values (*F*_ST_), and the upper triangle represents gene flow between pairwise regions, Table S13: Molecular variance analysis (AMOVA) of *M. micrantha*, Table S14: The position of outlier SNPs identified by BayeScan on the *M. micrantha* genome, Table S15: The annotation of the genes containing outlier SNPs, Table S16: Outlier SNPs associated with environmental variables identified using LFMM, Table S17: Outlier SNPs associated with environmental variables identified using BAYENV, Table S18: Gene family cluster analysis results of the genomes of *M. micrantha* and 12 other plants, Table S19: Gene families unique to *M. micrantha* and gene length distribution, Table S20: Protein sequences of 683 gene family members unique to *M. Micrantha*, Table S21: Kyoto Encyclopedia of Genes and Genomes (KEGG) pathway enrichment analysis of 683 gene families unique to *M. micrantha*, Table S22: Nonsynonymous amino acid mutation information and functional annotation of genes unique to *M. micrantha*, Table S23: Likelihood ratio test (LRT) of the variable w ratio under different models, Table S24: The annotation of the 41 positive selection genes in *M. micrantha*, Table S25: Kyoto Encyclopedia of Genes and Genomes (KEGG) pathway enrichment analysis of 41 positive selection genes in *M. micrantha*.

## References

[1] Seebens H., Blackburn T.M., Dyer E.E., Genovesi P., Hulme P.E., Jeschke J.M., Pagad S., Pysek P., Winter M., Arianoutsou M. (2017). No saturation in the accumulation of alien species worldwide. Nat. Commun..

[2] Briski E., Chan F.T., Darling J.A., Lauringson V., MacIsaac H.J., Zhan A., Bailey S.A. (2018). Beyond propagule pressure: Importance of selection during the transport stage of biological invasions. Front. Ecol. Environ..

[3] Bock D.G., Caseys C., Cousens R.D., Hahn M.A., Heredia S.M., Hubner S., Turner K.G., Whitney K.D., Rieseberg L.H. (2015). What we still don’t know about invasion genetics. Mol. Ecol..

[4] Van Boheemen L.A., Lombaert E., Nurkowski K.A., Gauffre B., Rieseberg L.H., Hodgins K.A. (2017). Multiple introductions, admixture and bridgehead invasion characterize the introduction history of *Ambrosia artemisiifolia* in Europe and Australia. Mol. Ecol..

[5] Brandes U., Furevik B.B., Nielsen L.R., Kjær E.D., Rosef L., Fjellheim S., Zhan A. (2019). Introduction history and population genetics of intracontinental scotch broom (*Cytisus scoparius*) invasion. Divers. Distrib..

[6] Smith A.L., Hodkinson T.R., Villellas J., Catford J.A., Csergo A.M., Blomberg S.P., Crone E.E., Ehrlen J., Garcia M.B., Laine A.-L. (2020). Global gene flow releases invasive plants from environmental constraints on genetic diversity. Proc. Natl. Acad. Sci. USA.

[7] Eckert C.G., Samis K.E., Lougheed S.C. (2008). Genetic variation across species’ geographical ranges: The central-marginal hypothesis and beyond. Mol. Ecol..

[8] Keller S.R., Taylor D.R. (2010). Genomic admixture increases fitness during a biological invasion. J. Evol. Biol..

[9] Qiao H., Liu W., Zhang Y., Zhang Y.-Y., Li Q.Q. (2019). Genetic admixture accelerates invasion via provisioning rapid adaptive evolution. Mol. Ecol..

[10] Prentis P.J., Wilson J.R.U., Dormontt E.E., Richardson D.M., Lowe A.J. (2008). Adaptive evolution in invasive species. Trends Plant Sci..

[11] Lee C.E. (2002). Evolutionary genetics of invasive species. Trends Ecol. Evol..

[12] Moran E.V., Alexander J.M. (2014). Evolutionary responses to global change: Lessons from invasive species. Ecol. Lett..

[13] Chen C., Liu Z., Pan Q., Chen X., Wang H., Guo H., Liu S., Lu H., Tian S., Li R. (2016). Genomic analyses reveal demographic history and temperate adaptation of the newly discovered honey bee subspecies *Apis mellifera sinisxinyuan* n. ssp. Mol. Biol. Evol..

[14] Hodgins K.A., Lai Z., Nurkowski K., Huang J., Rieseberg L.H. (2013). The molecular basis of invasiveness: Differences in gene expression of native and introduced common ragweed (*Ambrosia artemisiifolia*) in stressful and benign environments. Mol. Ecol..

[15] Van Boheemen L.A., Hodgins K.A. (2020). Rapid repeatable phenotypic and genomic adaptation following multiple introductions. Mol. Ecol..

[16] Davey J.W., Hohenlohe P.A., Etter P.D., Boone J.Q., Catchen J.M., Blaxter M.L. (2011). Genome-wide genetic marker discovery and genotyping using next-generation sequencing. Nat. Rev. Genet..

[17] Elshire R.J., Glaubitz J.C., Sun Q., Poland J.A., Kawamoto K., Buckler E.S., Mitchell S.E. (2011). A robust, simple genotyping-by-sequencing (GBS) approach for high diversity species. PLoS ONE.

[18] Martin M.D., Olsen M.T., Samaniego J.A., Zimmer E.A., Gilbert M.T.P. (2016). The population genomic basis of geographic differentiation in North American common ragweed (*Ambrosia artemisiifolia* L.). Ecol. Evol..

[19] Holm L.G., Plucknett D.L., Pancho J.V., Herberger J.P. (1977). The World’s Worst Weeds.

[20] Bhatt J.R., Singh J.S., Singh S.P., Tripathi R.S., Kohli R.K. (2012). Biology of *Mikania micrantha* H.B.K.: A Review. Invasive Alien Plants: An Ecological Appraisal for the Indian Subcontinent.

[21] Zhang L.Y., Ye W.H., Cao H.L., Feng H.L. (2004). *Mikania micrantha* H.B.K. in China—An overview. Weed Res..

[22] Dong L., Wu L.F. (2011). New research progress of *Mikania micrantha* H.B.K. J. Anhui Agric. Sci..

[23] Banerjee A.K., Mukherjee A., Dewanji A. (2017). Potential distribution of *Mikania micrantha* Kunth in India—Evidence of climatic niche and biome shifts. Flora.

[24] Yue M., Yu H., Li W., Yin A., Cui Y., Tian X. (2019). Flooding with shallow water promotes the invasiveness of *Mikania micrantha*. Ecol. Evol..

[25] Banerjee A.K., Mukherjee A., Guo W., Liu Y., Huang Y. (2019). Spatio-temporal patterns of climatic niche dynamics of an invasive plant Mikania micrantha Kunth and its potential distribution under projected climate change. Front. Ecol. Evol..

[26] Deng X. (2010). Morphological and physiological plasticity responding to different light environments of the invasive plant, *Mikania micrantha* H.B.Kunth. Ecol. Environ. Sci..

[27] Banerjee A.K., Mukherjee A., Guo W., Ng W.L., Huang Y. (2019). Combining ecological niche modeling with genetic lineage information to predict potential distribution of *Mikania micrantha* Kunth in South and Southeast Asia under predicted climate change. Glob. Ecol. Conserv..

[28] Guo Q., Qiang S., Lin J., Yu Y. (2005). The biological characteristics and integrated management of *Mikania micrantha*. Wuyi Sci. J..

[29] Wang B., Liao W., Zan Q., Li M., Zhou X., Gao S. (2003). The spreads of *Mikania micrantha* in China. Acta Sci. Nat. Univ. Sunyatseni.

[30] Wang T., Su Y., Chen G. (2008). Population genetic variation and structure of the invasive weed *Mikania micrantha* in southern China: Consequences of rapid range expansion. J. Hered..

[31] Wang T., Chen G., Zan Q., Wang C., Su Y. (2012). AFLP genome scan to detect genetic structure and candidate loci under selection for local adaptation of the invasive weed *Mikania micrantha*. PLoS ONE.

[32] Geng S.L., Chen Q., Cai W.L., Cao A.C., Ou-Yang C.B. (2017). Genetic variation in the invasive weed *Mikania micrantha* (Asteraceae) suggests highways as corridors for its dispersal in southern China. Ann. Bot..

[33] Banerjee A.K., Hou Z., Lin Y., Lan W., Tan F., Xing F., Li G., Guo W., Huang Y. (2020). Going with the flow: Analysis of population structure reveals high gene flow shaping invasion pattern and inducing range expansion of *Mikania micrantha* in Asia. Ann. Bot..

[34] Wang T., Wang Z., Chen G., Wang C., Su Y. (2016). Invasive chloroplast population genetics of *Mikania micrantha* in China: No local adaptation and negative correlation between diversity and geographic distance. Front. Plant Sci..

[35] Shen J., Wang Z., Su Y., Wang T. (2021). Associations between population epigenetic differentiation and environmental factors in the exotic weed mile-a-minute (*Mikania micrantha*). Weed Sci..

[36] Su Y.J., Wang T., Zheng B., Jiang Y., Chen G.P., Ouyang P.Y., Sun Y.F. (2005). Genetic differentiation of relictual populations of *Alsophila spinulosa* in southern China inferred from cpDNA *trnL-F* noncoding sequences. Mol. Phylogenet. Evol..

[37] Li H., Durbin R. (2009). Fast and accurate short read alignment with Burrows-Wheeler transform. Bioinformatics.

[38] Li H., Handsaker B., Wysoker A., Fennell T., Ruan J., Homer N., Marth G., Abecasis G., Durbin R., Subgroup G.P.D.P. (2009). The sequence alignment/map format and SAMtools. Bioinformatics.

[39] DePristo M.A., Banks E., Poplin R., Garimella K.V., Maguire J.R., Hartl C., Philippakis A.A., del Angel G., Rivas M.A., Hanna M. (2011). A framework for variation discovery and genotyping using next-generation DNA sequencing data. Nat. Genet..

[40] Wang K., Li M., Hakonarson H. (2010). ANNOVAR: Functional annotation of genetic variants from high-throughput sequencing data. Nucleic Acids Res..

[41] Goudet J. (2005). Hierfstat, a package for R to compute and test hierarchical *F*-statistics. Mol. Ecol. Notes.

[42] Pohlert T. The Pairwise Multiple Comparison of Mean Ranks Package (PMCMR). https://cran.r-project.org/web/packages/PMCMR/index.html.

[43] Kamvar Z.N., Tabima J.F., Grunwald N.J. (2014). *Poppr*: An R package for genetic analysis of populations with clonal, partially clonal, and/or sexual reproduction. PeerJ.

[44] Keenan K., McGinnity P., Cross T.F., Crozier W.W., Prodöhl P.A. (2013). diveRsity: An R package for the estimation and exploration of population genetics parameters and their associated errors. Methods Ecol. Evol..

[45] Stéphane D., Anne-Béatrice D. (2007). The ade4 package: Implementing the duality diagram for ecologists. J. Stat. Softw..

[46] Purcell S., Neale B., Todd-Brown K., Thomas L., Ferreira M.A.R., Bender D., Maller J., Sklar P., de Bakker P.I.W., Daly M.J. (2007). PLINK: A tool set for whole-genome association and population-based linkage analyses. Am. J. Hum. Genet..

[47] Alexander D.H., Lange K. (2011). Enhancements to the ADMIXTURE algorithm for individual ancestry estimation. BMC Bioinform..

[48] Yang J., Lee S.H., Goddard M.E., Visscher P.M. (2011). GCTA: A tool for genome-wide complex trait analysis. Am. J. Hum. Genet..

[49] Tamura K., Peterson D., Peterson N., Stecher G., Nei M., Kumar S. (2011). MEGA5: Molecular evolutionary genetics analysis using maximum likelihood, evolutionary distance, and maximum parsimony methods. Mol. Biol. Evol..

[50] Rambaut A. FigTree v1.4.2: Tree Figure Drawing Tool. http://tree.bio.ed.ac.uk/software/figtree/.

[51] Huete A., Didan K., Miura T., Rodriguez E.P., Gao X., Ferreira L.G. (2002). Overview of the radiometric and biophysical performance of the MODIS vegetation indices. Remote Sens. Environ..

[52] Naimi B., Hamm N.A.S., Groen T.A., Skidmore A.K., Toxopeus A.G. (2014). Where is positional uncertainty a problem for species distribution modelling?. Ecography.

[53] Foll M., Gaggiotti O. (2008). A genome-scan method to identify selected loci appropriate for both dominant and codominant markers: A Bayesian perspective. Genetics.

[54] Lotterhos K.E., Whitlock M.C. (2014). Evaluation of demographic history and neutral parameterization on the performance of *F*_ST_ outlier tests. Mol. Ecol..

[55] Eddy S.R. (1998). Profile hidden Markov models. Bioinformatics.

[56] Conesa A., Götz S., García-Gómez J.M., Terol J., Talón M., Robles M. (2005). Blast2GO: A universal tool for annotation, visualization and analysis in functional genomics research. Bioinformatics.

[57] Buchfink B., Xie C., Huson D.H. (2015). Fast and sensitive protein alignment using DIAMOND. Nat. Methods.

[58] Frichot E., François O. (2015). LEA: An R package for landscape and ecological association studies. Methods Ecol. Evol..

[59] Frichot E., Schoville S.D., Bouchard G., Francois O. (2013). Testing for associations between loci and environmental gradients using latent factor mixed models. Mol. Biol. Evol..

[60] Günther T., Coop G. A short manual for BayEnv2. https://bitbucket.org/tguenther/bayenv2_public/src/default/bayenv2_manual.pdf.

[61] Li L., Stoeckert C.J., Roos D.S. (2003). OrthoMCL: Identification of ortholog groups for eukaryotic genomes. Genome Res..

[62] Mao X., Cai T., Olyarchuk J.G., Wei L. (2005). Automated genome annotation and pathway identification using the KEGG Orthology (KO) as a controlled vocabulary. Bioinformatics.

[63] Edgar R.C. (2004). MUSCLE: Multiple sequence alignment with high accuracy and high throughput. Nucleic Acids Res..

[64] Yang Z. (2007). PAML 4: Phylogenetic analysis by maximum likelihood. Mol. Biol. Evol..

[65] Lucardi R.D., Wallace L.E., Ervin G.N. (2017). Invasion success in cogongrass (*Imperata cylindrica*): A population genetic approach exploring genetic diversity and historical iIntroductions. Invasive Plant Sci. Manag..

[66] Kalb D.M., Delaney D.A., DeYoung R.W., Bowman J.L. (2019). Genetic diversity and demographic history of introduced sika deer on the Delmarva Peninsula. Ecol. Evol..

[67] Allendorf F.W. (1986). Genetic drift and the loss of alleles versus heterozygosity. Zoo Biol..

[68] Xia L., Geng Q., An S. (2020). Rapid genetic divergence of an invasive species, *Spartina alterniflora*, in China. Front. Genet..

[69] Yang M., He Z., Huang Y., Lu L., Yan Y., Hong L., Shen H., Liu Y., Guo Q., Jiang L. (2017). The emergence of the hyperinvasive vine, *Mikania micrantha* (Asteraceae), via admixture and founder events inferred from population transcriptomics. Mol. Ecol..

[70] Chun Y.J., Fumanal B., Laitung B., Bretagnolle F. (2010). Gene flow and population admixture as the primary post-invasion processes in common ragweed (*Ambrosia artemisiifolia*) populations in France. New Phytol..

[71] Hong L., Shen H., Ye W.H., Cao H.L., Wang Z.M. (2007). Self-incompatibility in *Mikania micrantha* in South China. Weed Res..

[72] Li M., Lu E., Guo Q., Zan Q., Wei P., Jiang L., Xu H., Zhong T. (2012). Evaluation of the controlling methods and strategies for *Mikania micrantha* H.B.K. Acta Ecol. Sinica.

[73] Guo Q.F. (2014). Central-marginal population dynamics in species invasions. Front. Ecol. Evol..

[74] Guo Q., Taper M., Schoeneberger M.M., Brandle J.R. (2005). Spatial temporal population dynamics across a species’ range: From center to margin. Oikos.

[75] Bravo-Monzón Á.E., González-Rodríguez A., Espinosa-García F.J. (2018). Spatial structure of genetic and chemical variation in native populations of the mile-a-minute weed *Mikania micrantha*. Biochem. Syst. Ecol..

[76] Winkler D.E., Chapin K.J., Francois O., Garmon J.D., Gaut B.S., Huxman T.E. (2019). Multiple introductions and population structure during the rapid expansion of the invasive Sahara mustard (*Brassica tournefortii*). Ecol. Evol..

[77] Vanden Broeck A., Van Landuyt W., Cox K., De Bruyn L., Gyselings R., Oostermeijer G., Valentin B., Bozic G., Dolinar B., Illyés Z. (2014). High levels of effective long-distance dispersal may blur ecotypic divergence in a rare terrestrial orchid. BMC Ecol..

[78] Choudhury M.R., Deb P., Singha H., Chakdar B., Medhi M. (2016). Predicting the probable distribution and threat of invasive *Mimosa diplotricha* Suavalle and *Mikania micrantha* Kunth in a protected tropical grassland. Ecol. Eng..

[79] Chen B., Su J., Liao H., Peng S. (2018). A greater foraging scale, not a higher foraging precision, may facilitate invasion by exotic plants in nutrient-heterogeneous conditions. Ann. Bot..

[80] Stitt M., Gibon Y., Lunn J.E., Piques M. (2007). Multilevel genomics analysis of carbon signalling during low carbon availability: Coordinating the supply and utilisation of carbon in a fluctuating environment. Funct. Plant Biol..

[81] McLaughlin J.E., Boyer J.S. (2004). Glucose localization in maize ovaries when kernel number decreases at low water potential and sucrose is fed to the stems. Ann. Bot..

[82] Valmonte G.R., Arthur K., Higgins C.M., MacDiarmid R.M. (2014). Calcium-dependent protein kinases in plants: Evolution, expression and function. Plant Cell Physiol..

[83] Hafsi C., Falleh H., Saada M., Ksouri R., Abdelly C. (2017). Potassium deficiency alters growth, photosynthetic performance, secondary metabolites content, and related antioxidant capacity in Sulla carnosa grown under moderate salinity. Plant Physiol. Bioch..

[84] Sardans J., Peñuelas J. (2015). Potassium: A neglected nutrient in global change. Glob. Ecol. Biogeogr..

[85] Ou Q., Yang Y., Liang W., Sun F., Peng C. (2020). Effects of leaf leachates of the invasive plant *Mikania micrantha* H.B.K. on soil potassium activation and soil enzyme activity. J. South China Norm. Univ. (Nat. Sci. Ed.).

[86] Cooke J., Leishman M.R. (2016). Consistent alleviation of abiotic stress with silicon addition: A meta-analysis. Funct. Ecol..

[87] Zhou Y., Su Y., Zhong Y., Xie P., Xu M., Su Z. (2019). Community attributes predict the relationship between habitat invasibility and land use types in an agricultural and forest landscape. Forests.

[88] Shen Q., Zhang L., Liao Z., Wang S., Yan T., Shi P., Liu M., Fu X., Pan Q., Wang Y. (2018). The genome of *Artemisia annua* provides insight into the evolution of Asteraceae family and artemisinin biosynthesis. Mol. Plant.

[89] Song C., Liu Y., Song A., Dong G., Zhao H., Sun W., Ramakrishnan S., Wang Y., Wang S., Li T. (2018). The *Chrysanthemum nankingense* genome provides insights into the evolution and diversification of *Chrysanthemum* flowers and medicinal traits. Mol. Plant.

[90] Tian D., Pan X., Yu Y., Wang W., Zhang F., Ge Y., Shen X., Shen F., Liu X. (2013). De novo characterization of the *Anthurium* transcriptome and analysis of its digital gene expression under cold stress. BMC Genom..

[91] He M., Qin C., Wang X., Ding N. (2020). Plant unsaturated fatty acids: Biosynthesis and regulation. Front. Plant Sci..

[92] Cui C., Wang Z., Su Y., Wang T. (2021). New insight into the rapid growth of the *Mikania micrantha* stem based on DIA proteomic and RNA-Seq analysis. J. Proteom..

[93] Wu M., Li Z., Wang J. (2020). Transcriptional analyses reveal the molecular mechanism governing shade tolerance in the invasive plant *Solidago canadensis*. Ecol. Evol..

[94] Azzouz-Olden F., Hunt A.G., Dinkins R. (2020). Transcriptome analysis of drought-tolerant sorghum genotype SC56 in response to water stress reveals an oxidative stress defense strategy. Mol. Biol. Rep..

[95] Peters L.P., Carvalho G., Vilhena M.B., Creste S., Azevedo R.A., Monteiro-Vitorello C.B. (2017). Functional analysis of oxidative burst in sugarcane smut-resistant and -susceptible genotypes. Planta.

[96] Shin S., Zheng P., Fazio G., Mazzola M., Main D., Zhu Y. (2016). Transcriptome changes specifically associated with apple (*Malus domestica*) root defense response during *Pythium ultimum* infection. Physiol. Mol. Plant Pathol..

[97] Qiu Q., Zhang G., Ma T., Qian W., Wang J., Ye Z., Cao C., Hu Q., Kim J., Larkin D.M. (2012). The yak genome and adaptation to life at high altitude. Nat. Genet..

[98] Zou C., Yu D. (2010). Analysis of the cold-responsive transcriptome in the mature pollen of *Arabidopsis*. J. Plant Biol..

[99] Ribeiro P.R., Willems L.A.J., Silva A.T., Fernandez L.G., de Castro R.D., Bucher J., Snoek B.L., Hilhorst H.W.M., Ligterink W. (2018). Transcriptome profiling of *Ricinus communis* L. provides new insights underlying the mechanisms towards thermotolerance during seed imbibition and germination. Ind. Crops Prod..

[100] Dang H.Q., Tran N.Q., Gill S.S., Tuteja R., Tuteja N. (2011). A single subunit MCM6 from pea promotes salinity stress tolerance without affecting yield. Plant Mol. Biol..

[101] Casati P., Walbot V. (2008). Maize lines expressing RNAi to chromatin remodeling factors are similarly hypersensitive to UV-B radiation but exhibit distinct transcriptome responses. Epigenetics.

[102] Goellner E.M., Putnam C.D., Kolodner R.D. (2015). Exonuclease 1-dependent and independent mismatch repair. DNA Repair.

[103] Huang G., Ma S., Bai L., Zhang L., Ma H., Jia P., Liu J., Zhong M., Guo Z. (2012). Signal transduction during cold, salt, and drought stresses in plants. Mol. Biol. Rep..

[104] Yuan H., Zeng X., Ling Z., Wei Z., Wang Y., Zhuang Z., Xu Q., Tang Y., Tashi N. (2017). Transcriptome profiles reveal cold acclimation and freezing tolerance of susceptible and tolerant hulless barley genotypes. Acta Physiol. Plant..

[105] Luo L., Kong X., Gao Z., Zheng Y., Yang Y., Li X., Yang D., Geng Y., Yang Y. (2020). Comparative transcriptome analysis reveals ecological adaption of cold tolerance in northward invasion of *Alternanthera philoxeroides*. BMC Genom..

[106] Sun X., Hu S., Wang X., Liu H., Zhou Y., Guan Q. (2021). De novo assembly of *Amorpha fruticosa* L. transcriptome in response to drought stress provides insight into the tolerance mechanisms. PeerJ.

[107] Su W., Ye C., Zhang Y., Hao S., Li Q.Q. (2019). Identification of putative key genes for coastal environments and cold adaptation in mangrove *Kandelia obovata* through transcriptome analysis. Sci. Total Environ..

[108] Zhao D., Shi Y., Senthilkumar H.A., Qiao Q., Wang Q., Shen Y., Hu G. (2019). Enriched networks ‘nucleoside/nucleotide and ribonucleoside/ribonucleotide metabolic processes’ and ‘response to stimulus’ potentially conferred to drought adaptation of the epiphytic orchid *Dendrobium wangliangii*. Physiol. Mol. Biol. Plants.

[109] Lu L., Chen Y., Lu L., Lu Y., Li L. (2015). Transcriptome analysis reveals dynamic changes in the gene expression of tobacco seedlings under low potassium stress. J. Genet..

[110] Nayak S.S., Pradhan S., Sahoo D., Parida A. (2020). De novo transcriptome assembly and analysis of *Phragmites karka*, an invasive halophyte, to study the mechanism of salinity stress tolerance. Sci. Rep..

[111] Swaminathan P., Ohrtman M., Carinder A., Deuja A., Wang C., Gaskin J., Fennell A., Clay S. (2020). Water deficit transcriptomic responses differ in the invasive *Tamarix chinensis* and *T. ramosissima* established in the southern and northern United States. Plants.

