# Supplementary material for: Population Genomics Reveals Gene Flow and Adaptive Signature in Invasive Weed Mikania micrantha
Source: Genes (Basel). 2021 Aug 20;12(8):1279. doi: 10.3390/genes12081279 (PMC8394975; doi:10.3390/genes12081279)
Supplement: Supplementary file 1 [file genes-12-01279-s001.zip › Supplementary Files.pdf]

Supplementary Files

Population Genomics Reveals Gene Flow and Adaptive Signature in Invasive Weed *Mikania micrantha*

Xiaoxian Ruan [1], Zhen Wang [1], Yingjuan Su [1,2,*] and Ting Wang [3,*]

1 School of Life Sciences, Sun Yat-sen University, Guangzhou 510275, China; ruanxx@mail2.sysu.edu.cn (X.R.); wangzh535@mail2.sysu.edu.cn (Z.W.); suyj@mail.sysu.edu.cn (Y.S.)
2 Research Institute of Sun Yat-sen University in Shenzhen, Shenzhen, 518057, China; suyj@mail.sysu.edu.cn (Y.S.)
3 College of Life Sciences, South China Agricultural University, Guangzhou 510641, China; tingwang@scau.edu.cn (T.W.)
* Correspondence: suyj@mail.sysu.edu.cn (Y.S.); tingwang@scau.edu.cn (T.W.); Tel.: +86-020-84111939 (Y.S.); +86-020-85280185 (T.W.)

Table S1. Sampling description of 306 *M. micrantha* individuals and soil samples from 21 invasive populations in southern China.

Region	Population ID	Longitude	Latitude	Individual Number	Soil Sample Number
Hongkong (HK)	HK1	114°2'33.06"E	22°29'21.5"N	16	3
	HK3	114°14'3.02"E	22°23'26.21"N	15	3
	HK4	114°15'38.21"E	22°31'36.59"N	15	3
	HK5	114°9'10.08"E	22°23'49.08"N	10	3
	HK6	114°11'48.65"E	22°15'24.81"N	15	3
	HK7	114°8'29.5"E	22°27'2.2"N	10	3
	HK8	114°8'3.29"E	22°23'11.94"N	10	3
Macao (MA)	MA1	113°33'20.24"E	22°8'25.31"N	15	3
	MA4	113°33'48.4"E	22°7'19.37"N	16	3
Shenzhen (SZ)	SZ1	114°03'28.87"E	22°33'12.96"N	16	3
	SZ4	114°02'12.78"E	22°34'26.71"N	15	3
	SZ5	114°10'10.72"E	22°34'47.98"N	15	3
Neiling-ding (NLD)	NLD2	113°49'1"E	22°24'31.83"N	15	3
	NLD3	113°48'13.63"E	22°24'12.88"N	16	3
	NLD5	113°48'0.26"E	22°25'13.61"N	15	3
	NLD6	113°48'43.34"E	22°24'55.5"N	16	3
Zhuhai (ZH)	ZH1	113°37'51.46"E	22°25'41.90"N	15	3
	ZH2	113°38'14.61"E	22°24'50.66"N	16	3
Dongguan (DG)	DG2	113°48'0.17"E	22°52'45.75"N	15	3
	DG3	113°46'5.33"E	22°54'17.22"N	15	3
	DG4	113°47'33.93"E	22°51'15.33"N	15	3

Table S8. Summary of transition and transversion mutations of single nucleotide polymorphisms (SNPs) identified across 306 *M. micrantha* individuals.

Transition/Transversion mutation model	SNP number
Mutation between bases A and G	5051
Mutation between bases C and T	5156
Mutation between bases A and C	1808
Mutation between bases A and T	2008
Mutation between bases T and G	1832
Mutation between bases C and G	1089

Table S9. The locations and mutation types of SNPs in the *M. micrantha* genome.

Category	Number of SNPs
Upstream	312
Downstream	316
Upstream/Downstream	21
Intronic	1448
Splicing	6
Intergenic	13,984
5′-UTR	62
3′-UTR	81
Synonymous	402
Nonsynonymous	305
unknown	4
Other	3

Upstream: SNP is located in the 1000 bp region upstream of the gene; Downstream: SNP is located in the 1000 bp region downstream of the gene; Upstream/Downstream: SNP is located in the 1000 bp region upstream of the gene and also in the 1000 bp region downstream of another gene; Intergenic: SNPs are located in intergenic regions; Intronic: SNPs are located in intronic regions; Splicing: SNPs are located at the splice site; 5′-UTR: SNPs are located in 5′ untranslated regions; 3′-UTR: SNPs are located in 3′ untranslated regions; Synonymous: synonymous amino acid substitution; Nonsynonymous: nonsynonymous amino acid substitution.

Table S10. The analysis results of the differences in genetic diversity parameters among the six regions obtained from Kruskal-Wallis and post hoc Nemenyi test.

Genetic parameters	Degrees of freedom	Chi-squared	*p*-value
Allelic richness (A_R)	5	9.750	0.083
Observed heterozygosity (H_O)	5	5.727	0.334
Gene diversity (H_S)	5	5.220	0.390
Inbreeding coefficient (F_{IS})	5	3.185	0.672

Table S11. Genetic differentiation and gene flow between pairwise populations for *M. micrantha*. The lower triangle represents genetic differentiation values (F_{ST}), and the upper triangle represents gene flow between pairwise populations.

Population	HK1	HK3	HK4	HK5	HK6	HK7	HK8	SZ1	SZ4	SZ5	DG2	DG3	DG4	NLD2	NLD3	NLD5	NLD6	ZH1	ZH2	MA1	MA4
HK1	-	3.426	4.38	8.083	2.875	10.62	6.893	15.375	8.083	14.456	13.639	22.477	14.456	4.295	4.467	6.329	5.848	5.702	8.679	3.656	6.893
HK3	0.068	-	4.852	5.432	1.943	3.987	5.432	3.596	2.836	3.782	3.917	4.295	2.762	1.63	1.962	2.438	2.225	2.497	3.083	1.905	2.467
HK4	0.054	0.049	-	5.306	2.086	4.958	4.214	5.702	3.271	4.652	6.507	5.185	4.214	2.354	2.327	3.039	3.271	3.373	4.136	2.25	3.373
HK5	0.03	0.044	0.045	-	2.875	9.75	14.456	7.563	6.694	10.167	10.167	7.563	5.702	2.799	2.691	3.538	3.848	3.538	4.38	2.201	3.321
HK6	0.08	0.114	0.107	0.08	-	2.591	2.875	3.426	3.481	2.657	3.596	2.691	2.762	3.128	3.128	4.214	4.136	3.596	3.656	2.177	2.624
HK7	0.023	0.059	0.048	0.025	0.088	-	7.103	7.326	6.694	10.62	9.365	9.009	8.083	2.762	3.222	4.295	3.656	3.917	5.564	2.875	3.538
HK8	0.035	0.044	0.056	0.017	0.08	0.034	-	9.365	4.852	9.009	8.371	7.326	5.564	2.799	2.657	3.782	3.596	3.718	4.75	2.528	3.271
SZ1	0.016	0.065	0.042	0.032	0.068	0.033	0.026	-	9.75	12.25	49.75	12.908	15.375	4.852	4.06	5.432	6.893	6.694	7.326	3.222	5.848
SZ4	0.03	0.081	0.071	0.036	0.067	0.036	0.049	0.025	-	6	8.371	6.329	7.563	3.083	3.175	4.295	4.467	4.467	4.467	2.41	4.295
SZ5	0.017	0.062	0.051	0.024	0.086	0.023	0.027	0.02	0.04	-	12.908	9.009	8.083	2.915	2.997	4.214	3.596	4.295	5.306	2.997	3.373
DG2	0.018	0.06	0.037	0.024	0.065	0.026	0.029	0.005	0.029	0.019	-	12.908	12.908	4.958	4.558	6.694	8.083	6.329	8.679	3.271	5.702
DG3	0.011	0.055	0.046	0.032	0.085	0.027	0.033	0.019	0.038	0.027	0.019	-	15.375	3.656	3.917	6.16	5.185	5.848	7.326	3.481	6
DG4	0.017	0.083	0.056	0.042	0.083	0.03	0.043	0.016	0.032	0.03	0.019	0.016	-	4.295	4.136	5.564	5.306	4.958	6.694	3.538	5.564
NLD2	0.055	0.133	0.096	0.082	0.074	0.083	0.082	0.049	0.075	0.079	0.048	0.064	0.055	-	17.607	7.103	62.25	8.083	9.365	2.836	4.558
NLD3	0.053	0.113	0.097	0.085	0.074	0.072	0.086	0.058	0.073	0.077	0.052	0.06	0.057	0.014	-	6.507	18.981	6	10.167	3.039	5.069
NLD5	0.038	0.093	0.076	0.066	0.056	0.055	0.062	0.044	0.055	0.056	0.036	0.039	0.043	0.034	0.037	-	10.62	9.009	17.607	3.782	6
NLD6	0.041	0.101	0.071	0.061	0.057	0.064	0.065	0.035	0.053	0.065	0.03	0.046	0.045	0.004	0.013	0.023	-	13.639	31	3.987	8.083
ZH1	0.042	0.091	0.069	0.066	0.065	0.06	0.063	0.036	0.053	0.055	0.038	0.041	0.048	0.03	0.04	0.027	0.018	-	16.417	3.917	6.16
ZH2	0.028	0.075	0.057	0.054	0.064	0.043	0.05	0.033	0.053	0.045	0.028	0.033	0.036	0.026	0.024	0.014	0.008	0.015	-	5.306	8.679
MA1	0.064	0.116	0.1	0.102	0.103	0.08	0.09	0.072	0.094	0.077	0.071	0.067	0.066	0.081	0.076	0.062	0.059	0.06	0.045	-	2.836
MA4	0.035	0.092	0.069	0.07	0.087	0.066	0.071	0.041	0.055	0.069	0.042	0.04	0.043	0.052	0.047	0.04	0.03	0.039	0.028	0.081	-

Table S12. Genetic differentiation and gene flow between pairwise regions for *M. micrantha*. The lower triangle represents genetic differentiation values (F_{ST}), and the upper triangle represents gene flow between pairwise regions.

Region	HK	SZ	DG	NLD	ZH	MA
HK	-	20.583	18.981	6	8.679	7.103
SZ	0.012	-	31	5.564	7.563	6.507
DG	0.013	0.008	-	6.893	9.009	9.009
NLD	0.04	0.043	0.035	-	22.477	9.009
ZH	0.028	0.032	0.027	0.011	-	14.456
MA	0.034	0.037	0.027	0.027	0.017	-

Table S13. Molecular variance analysis (AMOVA) of *M. micrantha*.

Source of variation	d.f.	Sum of squares	Variance components	Percentage of variation
Among populations	20	37176.946	35.624	4.16%
Within populations	591	485538.029	821.553	95.84%
Total	611	522714.975	857.178	100%
Fixation Index (F_{ST}): 0.042 p-value < 0.001				

d.f., degrees of freedom.

Table S16. Outlier SNPs associated with environmental variables identified using LFMM.

Chromosome	Position	Environmental variables			
Hic_asm_1	56244207	Bio12	Bio18		
Hic_asm_2	64422982	Bio12	C	Cr	PNV
Hic_asm_2	73718621	Bio12	Bio18		
Hic_asm_3	29391298	Bio18			
Hic_asm_6	5664974	Bio12			
Hic_asm_6	42111491	Bio18			
Hic_asm_6	79396270	PNV			
Hic_asm_7	44788339	C			
Hic_asm_8	1788483	Bio12	C		
Hic_asm_9	35305573	C			
Hic_asm_10	141801782	Bio12	Bio18		
Hic_asm_13	8640322	Bio18	Cr	Bio12	
Hic_asm_13	95869816	Bio12	C	PNV	
Hic_asm_14	3269220	Bio12			
Hic_asm_14	56405021	C			
Hic_asm_16	11549940	Bio18	PNV	Bio12	
Hic_asm_16	54017721	Bio12			
Hic_asm_16	65069126	K			

Bio12, annual precipitation; Bio18, precipitation of warmest quarter; PNV, percent of non-vegetation cover.

Table S17. Outlier SNPs associated with environmental variables identified using BAYENV.

Chromosome	Position	Environmental variables					
Hic_asm_2	59705425	Bio3	Bio9	Bio14			
Hic_asm_2	64422982	Bio3	Bio9				
Hic_asm_2	67009047	PTC					
Hic_asm_2	72037757	Bio3	Bio9	Bio14	C		
Hic_asm_2	73123514	Bio3	Bio9	Bio14	C	Si	
Hic_asm_2	74647226	Bio3	Bio9	Bio14			
Hic_asm_3	80329031	Bio3	Bio9	Bio14	Si		
Hic_asm_4	13971277	Bio3	Bio9	Bio14	C	Si	
Hic_asm_4	13971324	Bio3	Bio9	Bio14	C	Si	
Hic_asm_5	38926145	Ca					
Hic_asm_6	45893595	Bio14	C				
Hic_asm_6	79396270	C	Ca				
Hic_asm_7	42423247	Bio3	Bio9	Bio14	C		
Hic_asm_8	1788483	C					
Hic_asm_9	33697841	C					
Hic_asm_9	35305573	Bio3	Bio9	Bio14	C	Si	PNT
Hic_asm_10	16503156	Ca					
Hic_asm_10	84852364	C	Ca				
Hic_asm_14	2443805	Bio9	C				
Hic_asm_14	52856134	Bio3	Bio9	C			
Hic_asm_15	34256787	Bio3	Bio9	Bio14	C	Si	
Hic_asm_16	29030223	Bio3	Bio9	Bio14			
Hic_asm_17	13338860	C					
Hic_asm_17	15895453	Ca					

Bio3, isothermality; Bio9, mean temperature of driest quarter; Bio14, precipitation of driest month; PNT, percent of non-tree vegetation cover; PTC, percent of tree cover.

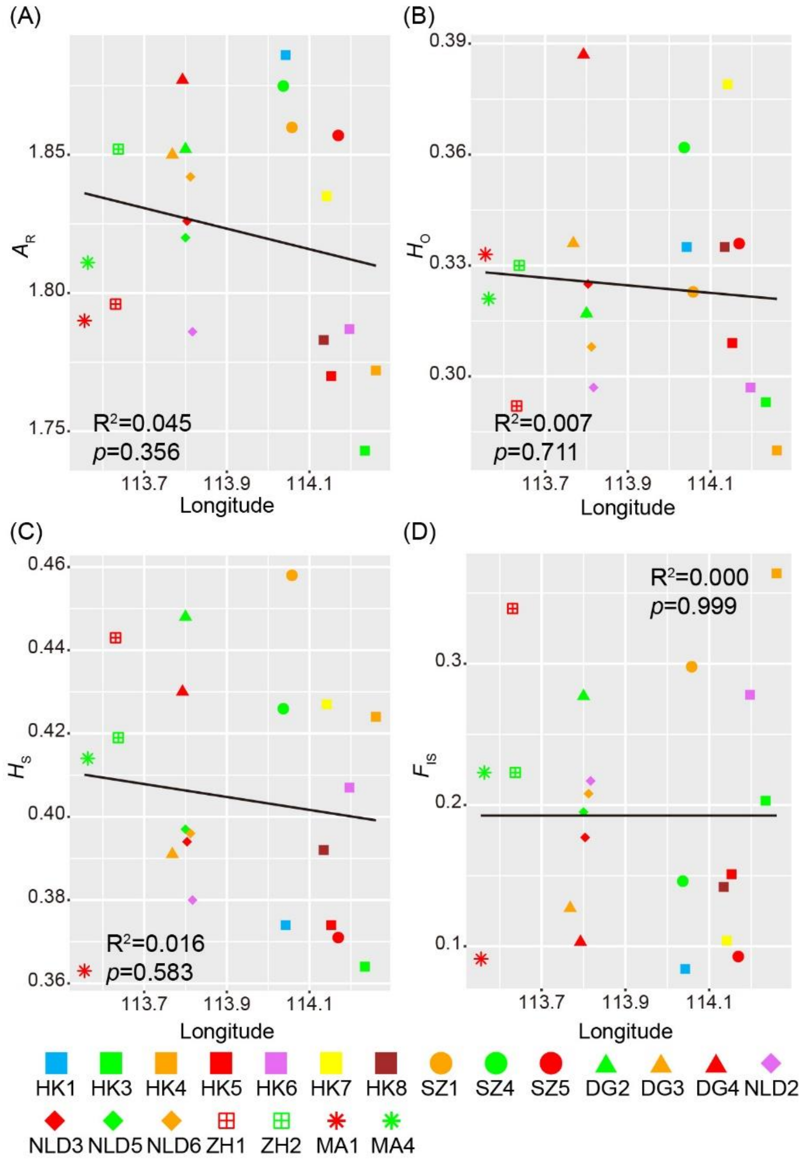

Figure S1. Correlation between genetic diversity indices and longitude of sampling populations. Diversity indices included allelic richness (A_R), observed heterozygosity (H_O), gene diversity (H_S), and inbreeding coefficient (F_{IS}).

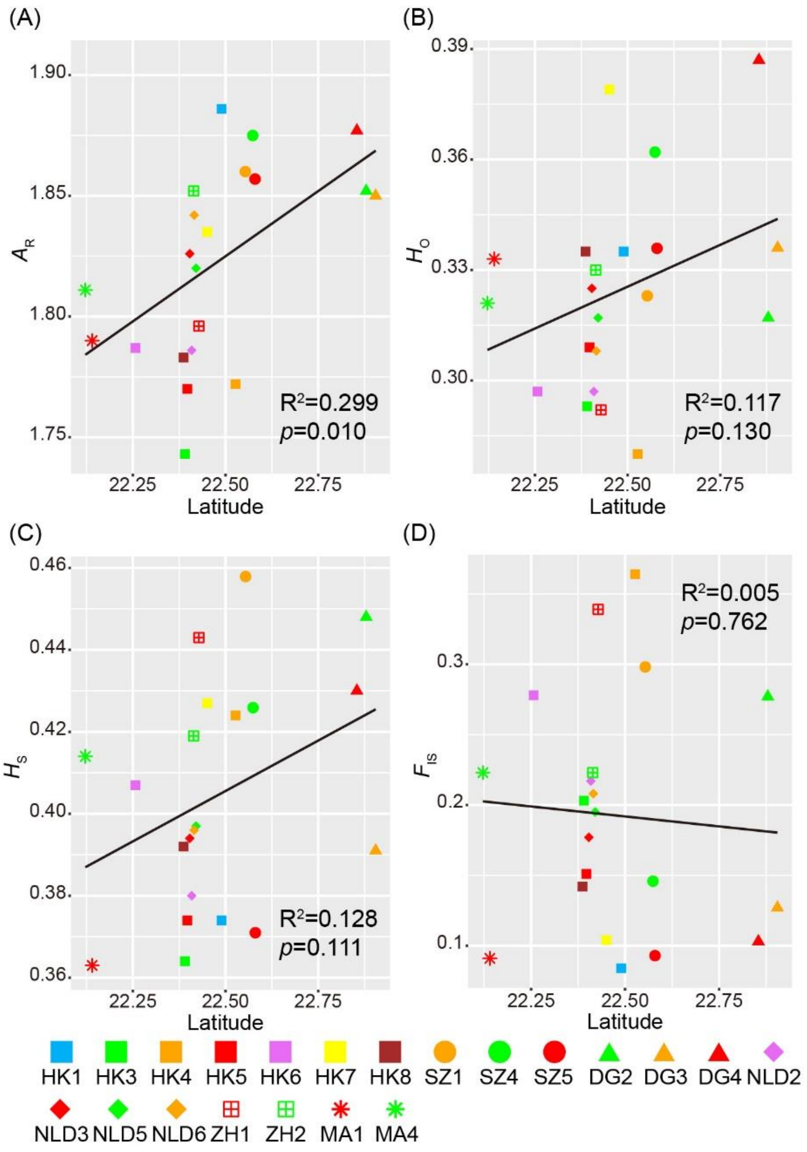

Figure S2. Correlation between genetic diversity indices and latitude of sampling populations. Diversity indices included allelic richness (A_R), observed heterozygosity (H_O), gene diversity (H_S), and inbreeding coefficient (F_{IS}).

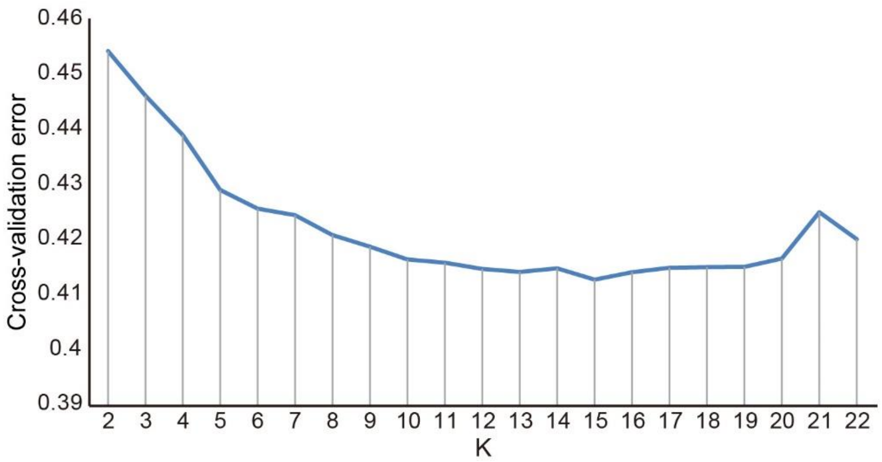

Figure S3. The optimum number of clusters (*K*) estimated with ADMIXTURE analysis based on cross-validation (CV) error.

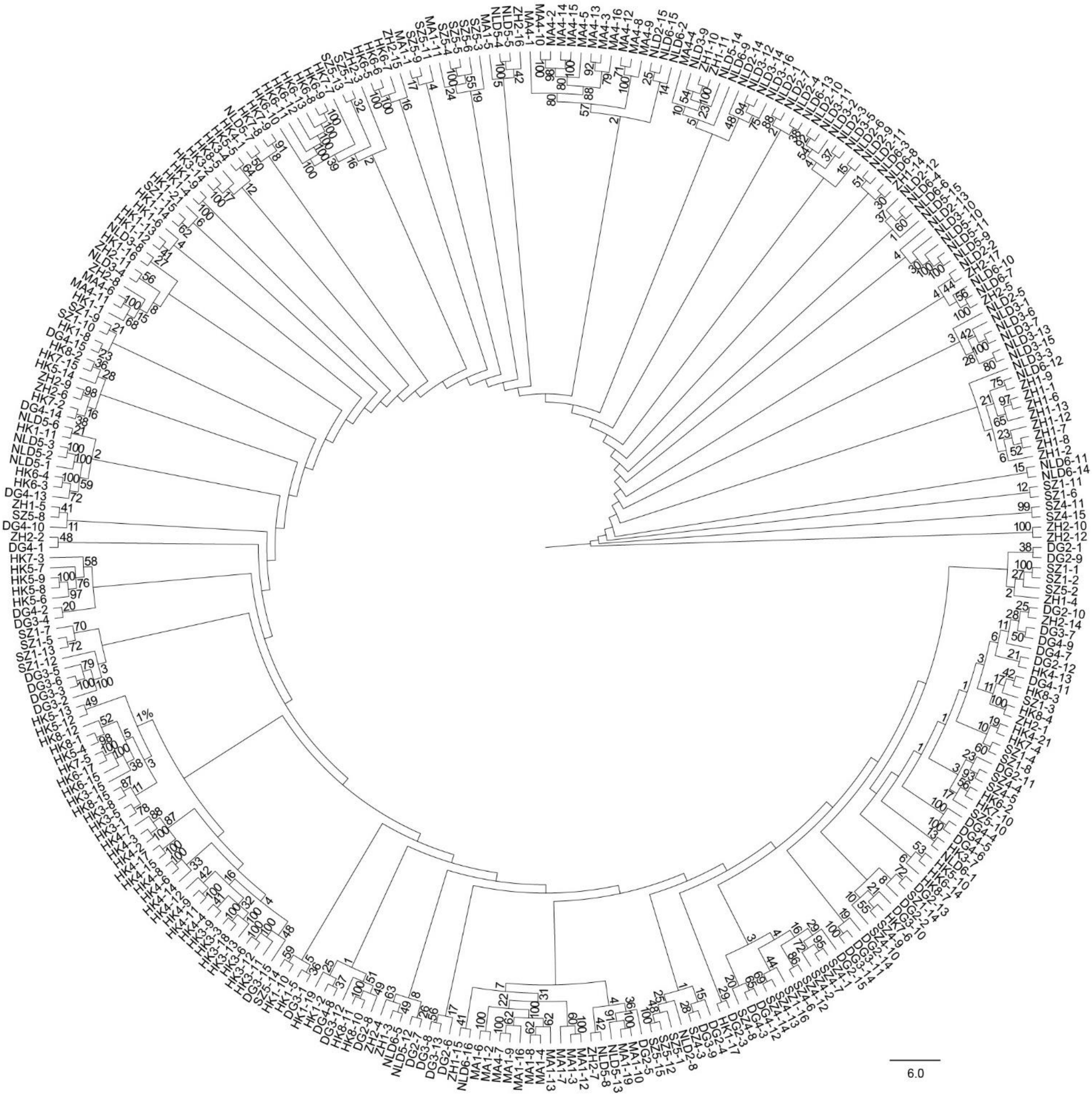

Figure S4. Unweight pair group method with arithmetic mean (UPGMA) tree constructed using the pairwise genetic distances among 306 *M. micrantha* individuals.

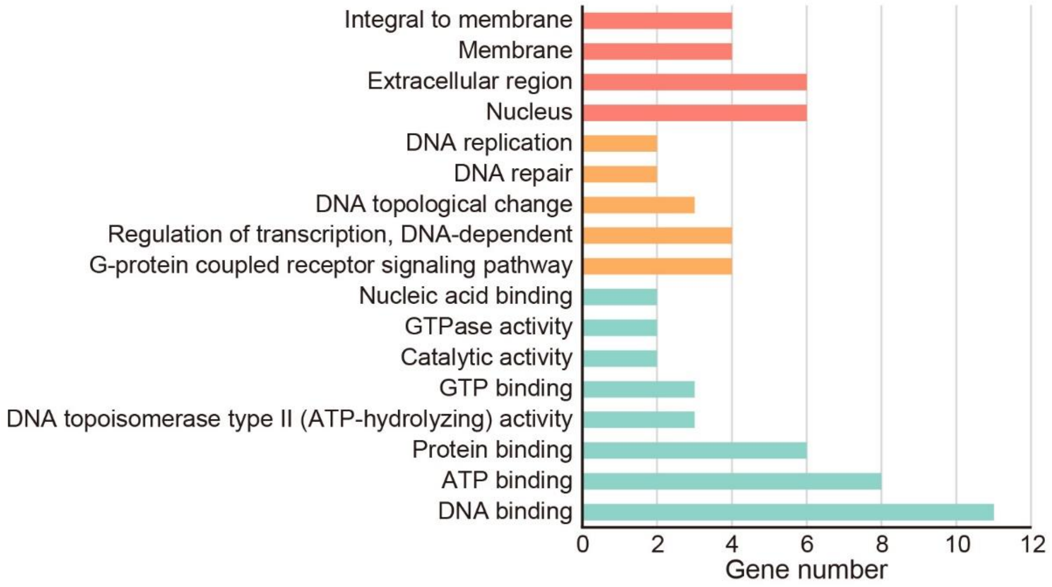

Figure S5. Gene ontology (GO) annotation of the positive selection genes in *M. micrantha*. The red, orange, and green bars represent cellular component, biological process, and molecular function categories, respectively.

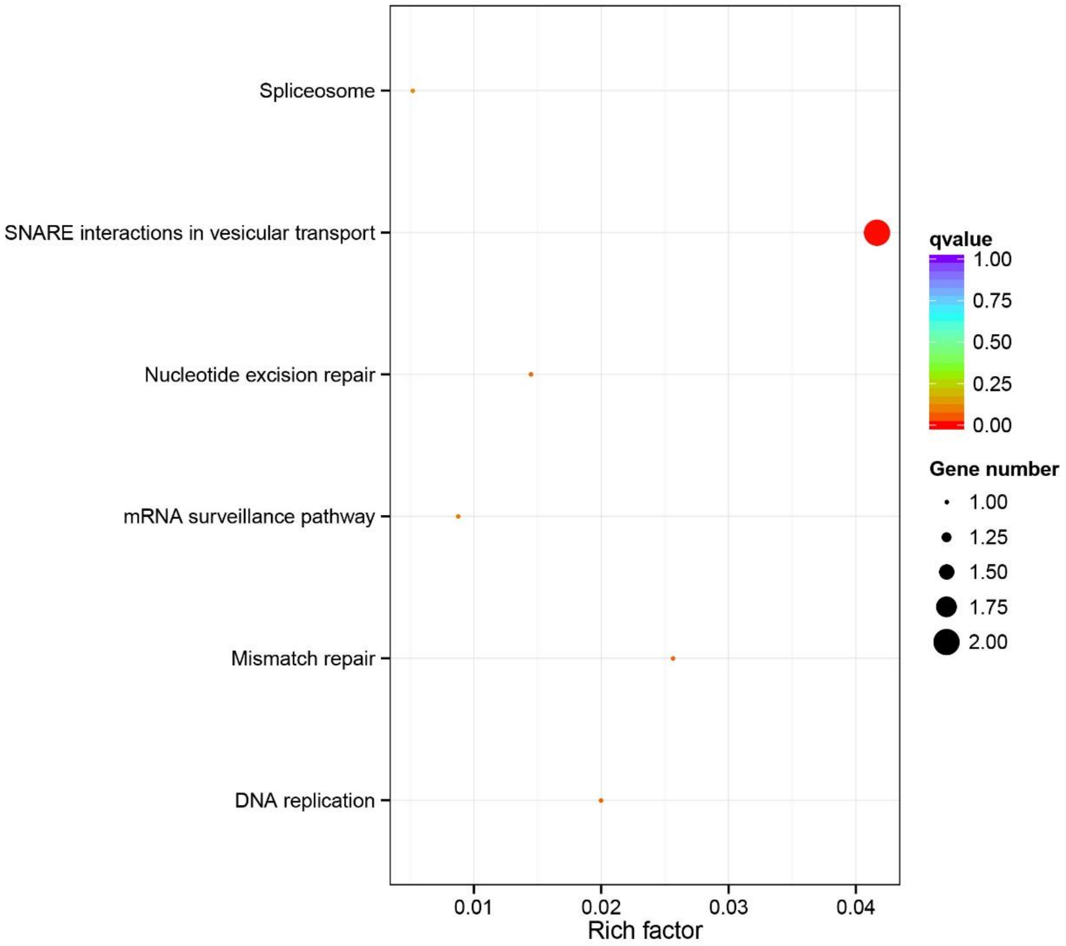

Figure S6. Kyoto Encyclopedia of Genes and Genomes (KEGG) pathway enrichment analysis of the positive selection genes in *M. micrantha*.
